# A Phase Separation‐Assisted Pre‐Enrichment Method for Ultrasensitive Respiratory Virus Detection

**DOI:** 10.1002/advs.202506578

**Published:** 2025-06-27

**Authors:** Yang Cao, Pui Ngan Lau, Alex W.H. Chin, Zhuolin He, Christina C. K. Au Yeung, Kehao Zeng, Haisong Lin, Leo L. M. Poon, Ho Cheung Shum

**Affiliations:** ^1^ Department of Mechanical Engineering The University of Hong Kong Pokfulam Road Hong Kong SAR 000000 China; ^2^ Advanced Biomedical Instrumentation Centre Hong Kong Science Park, Shatin, New Territories Hong Kong SAR 000000 China; ^3^ Division of Public Health Laboratory Sciences School of Public Health Li Ka Shing Faculty of Medicine The University of Hong Kong Hong Kong SAR 000000 China; ^4^ HKU‐Pasteur Research Pole School of Public Health Li Ka Shing Faculty of Medicine The University of Hong Kong Hong Kong SAR 000000 China; ^5^ Hong Kong Jockey Club Global Health Institute Li Ka Shing Faculty of Medicine The University of Hong Kong Hong Kong SAR 000000 China; ^6^ Weiyang College Tsinghua University Beijing 100084 China; ^7^ School of Engineering Westlake University Hangzhou 310030 China; ^8^ Research Center for Industries of the Future Westlake University Hangzhou 310030 China; ^9^ Department of Biomedical Engineering City University of Hong Kong Hong Kong SAR 000000 China; ^10^ Department of Chemistry City University of Hong Kong Hong Kong SAR 000000 China

**Keywords:** affinity‐driven partitioning, aqueous two‐phase systems, phase separation‐assisted preconcentration, point‐of‐care testing, respiratory virus detection

## Abstract

Enriching trace biomarkers (e.g., proteins, nucleic acids) is critical for biomedical applications; yet conventional methods often lack versatility, limiting their effectiveness to specific biomarker types. To address this, the phase separation‐assisted pre‐enrichment (PSAP) technology is presented that exploits differential polymer‐polymer partitioning to achieve 47‐fold antigen and 44‐fold RNA enrichment simultaneously. Through systematic optimization of interfacial chemistry, including pH modulation, polymer hydrophilicity, mass fraction, and molecular weights, the protocol is refined to enable direct integration with commercial diagnostics. PSAP‐boosted rapid antigen ests (RATs) detected SARS‐CoV‐2 and Influenza viruses at tenfold and fivefold lower limits, respectively. In clinical validation, 53 clinical specimens (containing PCR undetectable samples as controls) are analyzed. The PSAP method significantly enhanced detection accuracy for both viral antigens and RNA, particularly improving positivity rates in low viral load specimens (27 < Ct < 31) compared to conventional approaches while maintaining specificity in high‐Ct and negative controls. With its universality and tunability, PSAP demonstrates universal applicability across respiratory pathogens and lays the foundation for next‐generation point‐of‐care diagnostics.

## Graphical abstract

Conventional biomarker enrichment lacks versatility and specificity. Phase separation‐assisted pre‐enrichment (PSAP) is introduced for concurrent antigen (47×) and RNA (44×) enrichment. Tenfold and fivefold lower detection limits for SARS‐CoV‐2 and Influenza, respectively, were achieved. Enhanced sensitivity (Ct: 27–31) without cross‐reactivity was demonstrated in 53 clinical specimens. PSAP's universality positions it as transformative for point‐of‐care diagnostics.

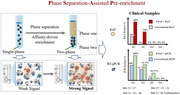

## 1 Introduction

Efficient enrichment of trace biomarkers – including proteins, nucleic acids, and metabolites – constitutes a cornerstone of precise detection.^[^ ^1^, ^2^ ^]^ While conventional enrichment techniques (e.g., filtration,^[^ ^3^ ^]^ magnetic separation,^[^ ^4^ ^]^ solvent evaporation^[^ ^5^ ^]^) have enabled biomarker analysis in controlled laboratory settings, their clinical utility and compatibility remain constrained by inherent limitations.^[^ ^6^ ^]^ Most existing methods demonstrate biomarker‐specific efficacy (e.g., the acetone crash method is commonly employed for proteins,^[^ ^7^ ^]^ although it is not widely utilized for nucleic acid extraction^[^ ^8^ ^]^), requiring customized protocols for different molecular classes that compromise workflow versatility. This technological bottleneck becomes particularly pronounced when handling complex biological matrices containing multiple biomarker species at ultralow concentrations.

Recent advances in point‐of‐care diagnostics have intensified the demand for universal pre‐enrichment strategies.^[^ ^9^ ^]^ For example, RATs, offering rapid analyte identification with user‐friendly operation and low implementation costs, have played an essential role in monitoring disease spread.^[^ ^10^, ^11^, ^12^ ^]^ However, insufficient viral protein concentration frequently leads to false‐negative results, particularly during early infection.^[^ ^13^, ^14^ ^]^ Similarly, even for gold‐standard quantitative reverse transcription polymerase chain reaction​ (RT‐qPCR), the analyses of low‐abundance nucleic acids in clinical specimens often require time‐consuming sample processing to achieve detectable thresholds.^[^ ^2^ ^]^ Thus, a robust pre‐enrichment technique that simultaneously concentrates diverse biomarkers can dramatically enhance detection sensitivity while reducing reliance on specialized instrumentation – two critical requirements for decentralized diagnostic applications.

Herein, we present a phase separation‐assisted pre‐enrichment (PSAP) strategy using tunable Aqueous Two‐Phase Systems (ATPSs).^[^ ^15^, ^16^, ^17^, ^18^, ^19^, ^20^ ^]^ Unlike membrane‐dependent separation methods,^[^ ^21^, ^22^, ^23^, ^24^ ^]^ PSAP exploits differential biomarker partitioning between immiscible phases, enabling > 47‐fold enrichment of viral antigens and > 44‐fold enrichment of viral RNA within 10 minutes, without target‐specific modifications and physical filtering steps. When applied to ​Severe Acute Respiratory Syndrome Coronavirus 2 (SARS‐CoV‐2) and Influenza A/B tests, PSAP boosted detection limits tenfold and fivefold in RATs, respectively. In clinical validation, 53 clinical specimens (including PCR‐negative controls) were analyzed to evaluate the PSAP method, which significantly improved detection accuracy for viral antigens and RNA, particularly increasing positivity rates in low viral load specimens (27 < Ct < 31) compared to conventional methods, while maintaining specificity in high‐Ct samples and negative controls. The method can be readily adapted for detecting diverse pathogens, including emerging variants, to bolster future pandemic preparedness.

## 2 Results and Discussions

### 2.1 Overview of PSAP Workflow

**Figure 1:**
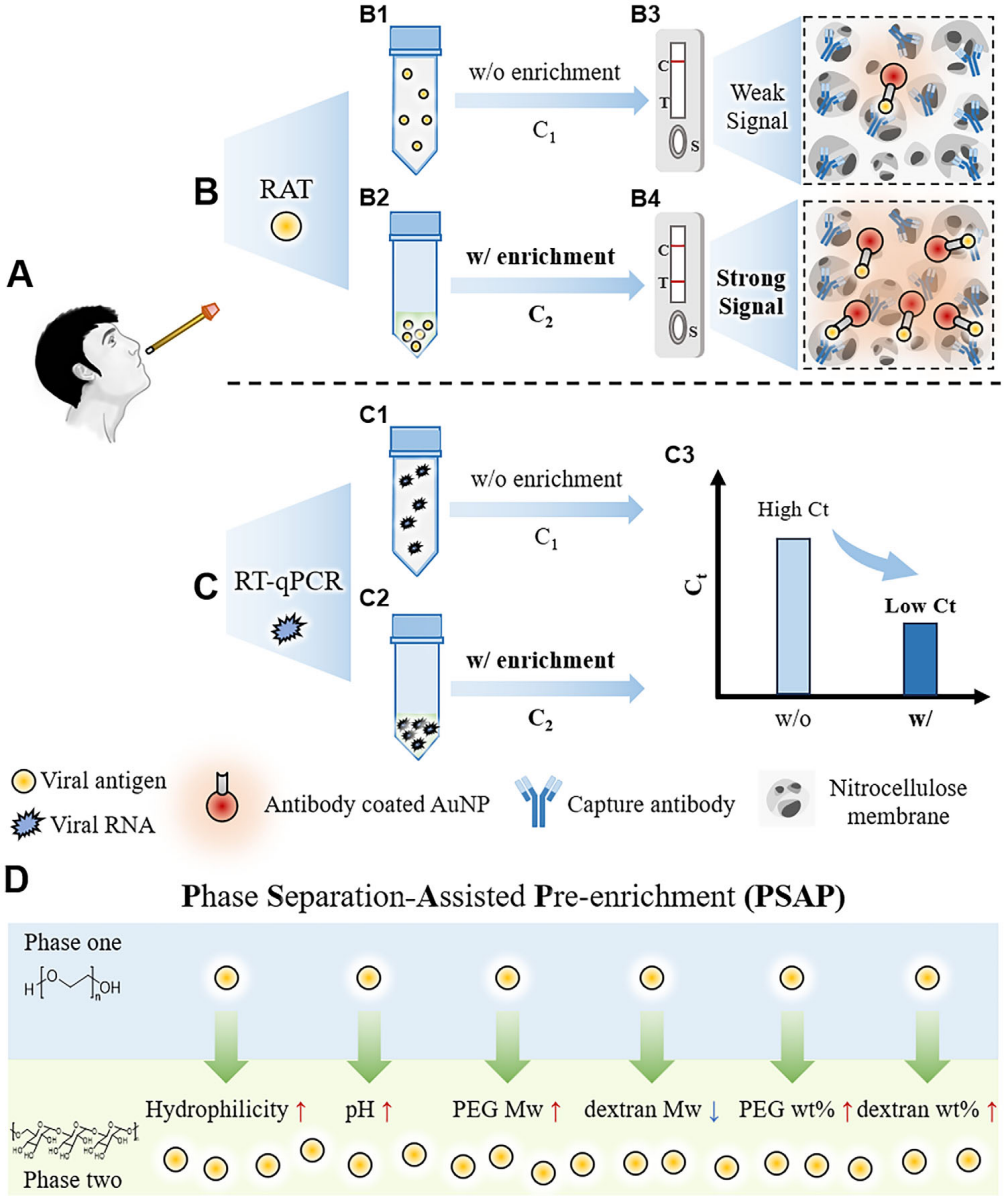
Overview of the PSAP technology for enriching viral antigen and RNA. A) The schematic illustrates that the viral samples are collected by nasopharyngeal swabs. B) Schematic of the conventional RATs and PSAP‐RATs. B1, B3: When the initial virus concentration in a single phase (C_1_) is below the limit of detection (LOD) of RATs, no signal can be observed at the test line. B2, B4: When the virus concentration in the bottom phase (C_2_) is enriched, a signal is generated at the test line. C) Schematic of enriching viral RNA and applying it to RT‐qPCR, as well as comparison to the conventional method. C3: A lower Ct value can be achieved with enrichment (C_2_) than without enrichment (C_1_), thus enabling the detection of low‐concentration samples. D) Schematic illustrating the enrichment mechanism via the affinity‐driven partitioning process. In this study, the influences on the partitioning affinity, induced by factors such as hydrophilicity, pH ranges, and the molecular weights and mass fractions of Polyethylene Glycol (PEG) and dextran, are evaluated.

The overall concept and performance of PSAP are introduced in **Figure** **1**. The viral samples are collected via nasal swabs first (Figure 1A). Then, unlike the conventional method of directly loading lysed samples to RATs (Figure 1B1), the PSAP method (Figure 1B2) utilizes the phase separation process to pre‐enrich the samples before conventional RATs. While the presence of low‐concentration antigens typically does not generate a signal (Figure 1B3), antigens that have been enriched through the PSAP method display clear signals (Figure 1B4). Meanwhile, conventional methods lacking viral RNA enrichment (Figure 1C1) exhibit detection limited to samples with low Ct values, whereas approaches incorporating enrichment (Figure 1C2) enable detection across a broader Ct range (Figure 1C3). Various factors can be tuned to control partitioning affinities (Figure 1D). In this work, five ATPSs parameters are investigated to improve the enrichment performance, including ATPSs types, buffer pH, molecular weights, mass ratios of ATPSs materials, and two‐phase volume ratios. In addition, the fluidity of polymer solutions is optimized to maintain the smooth flow of assays when applied to lateral flow strips.

### 2.2 Optimization of ATPSs for Antigen and RNA Enrichment

**Figure 2:**
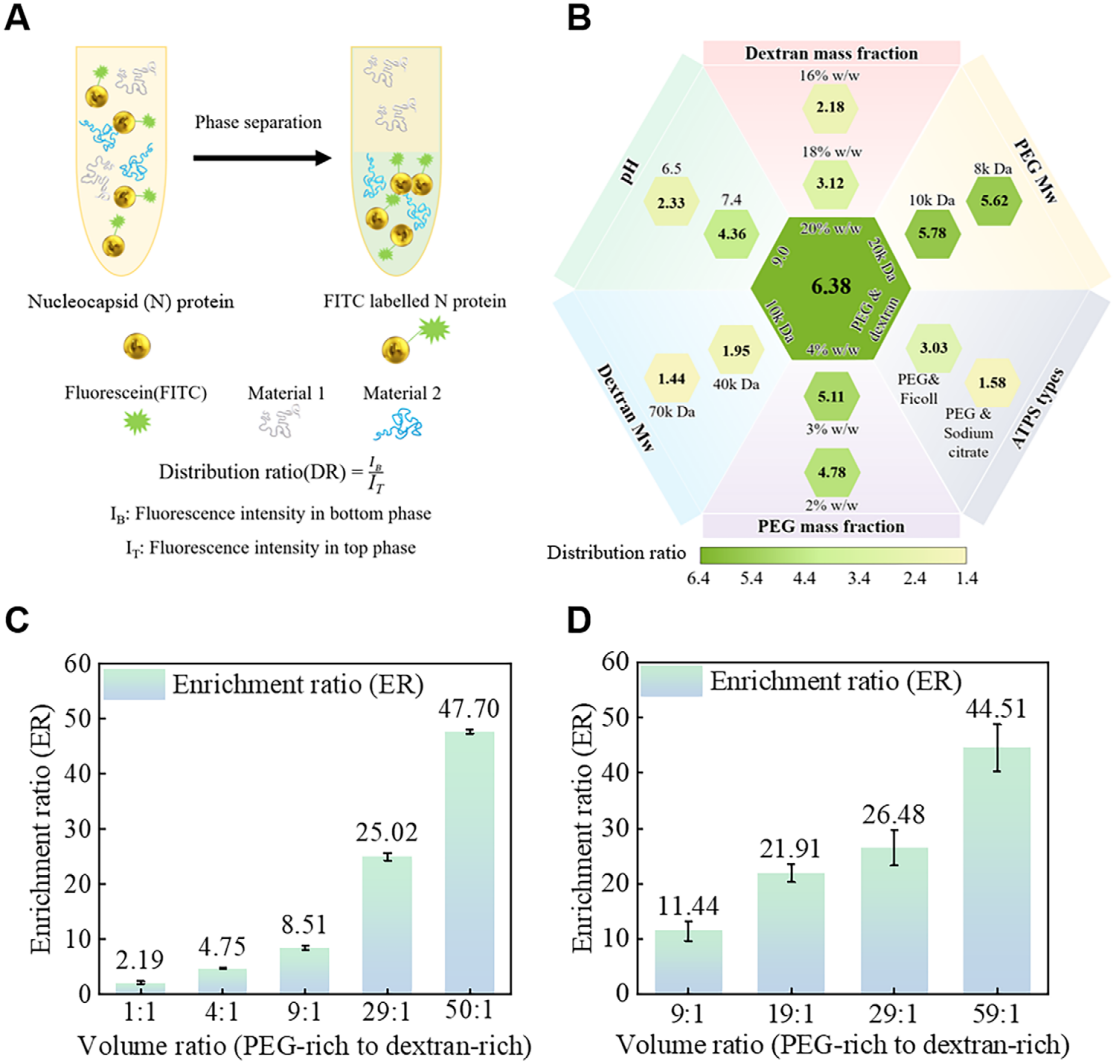
Modulation of antigen enrichment by screening compositions of ATPSs and the two‐phase volumetric ratios. A) Schematic of FITC‐N protein being partitioned into the dextran‐rich phase and definition of the DR. B) DR of antigens that are partitioned in different ATPSs. The ATPSs types, pH values, molecular weights, and mass fractions of PEG and dextran are investigated separately. The optimal composition parameter can be found in the innermost hexagon. C) Antigen enrichment ratios using different volume ratios. The enrichment ratio is calculated as the ratio of the enriched antigen concentration in the bottom phase to the initial value. The top‐to‐bottom volumetric ratios equal to 1:1, 4:1, 9:1, 29:1, and 50:1. D) Enrichment of viral RNA using different volume ratios. The enrichment ratio is calculated as the ratio of the enriched RNA concentration in the bottom phase to the initial concentration. The top‐to‐bottom volumetric ratios are equal to 9:1, 19:1, 29:1, and 59:1. The error bars represent the standard deviation from three independent tests.

The conceptual schematic (**Figure** **2**A) illustrates the process of target antigens being enriched into one of the two phases during phase separation. Various parameters can influence antigen partitioning performance. Here, we optimized major factors, including ATPSs types, buffer pH, polymer molecular weights, and polymer mass fractions in sequence (Figure 2B; Figure S1, Supporting Information). We compared the partitioning trends of Nucleocapsid (N) protein under various conditions by measuring the distribution ratio (DR).^[^ ^17^ ^]^ First, three types of ATPSs, PEG‐dextran, PEG‐Ficoll, and PEG‐salt, are investigated (Figure S1A, Supporting Information). Results indicated that choosing dextran as the bottom phase leads to higher antigen partitioning than Ficoll and sodium citrate. This is due to the surface hydrophilicity of N protein, which is caused by polar and charged amino acid residues that can interact with water molecules via hydrogen bonds.^[^ ^25^, ^26^ ^]^ Meanwhile, the two phases formed in ATPSs have different hydrophilicity and hydrophobicity.^[^ ^27^ ^]^ Consequently, the hydrophilic phase attracts the hydrophilic N protein more than the hydrophobic phase.^[^ ^28^ ^]^ Second, buffers with various pH values (pH 6.5, 7.4, and 9.0) are tested, and the higher pH is more favorable for concentrating N protein into the dextran‐rich phase (Figure S1B, Supporting Information). This phenomenon can be explained by the electrostatic interactions between the N protein and the dextran molecule. When buffer pH is lower than isoelectric point (9.5 ~10.5), the N protein is positively charged.^[^ ^21^ ^]^ At elevated pH values, dextran loses protons and acquires negative charges on its backbone, while the surface charge of PEG does not vary too much.^[^ ^29^, ^30^ ^]^ Consequently, the attractive interaction between N protein and dextran is strengthened, partitioning more N proteins into the dextran‐rich phase. Besides, the to‐be‐partitioned protein is more concentrated by smaller molecules and more repelled by larger polymer molecules when all other factors, such as polymer concentration, salt composition, temperature, and other factors, are kept constant.^[^ ^27^ ^]^ Consequently, it is observed that the antigen prefers to be in the bottom phase when the PEG molecular weight is increased, and the dextran molecular weight is decreased (Figure S1C,D, Supporting Information). Likewise, increasing dextran and decreasing PEG mass fractions resulted in a higher partitioning preference toward the bottom phase (Figure S1E,F, Supporting Information). The results of the tested parameters and their corresponding partitioning affinities are summarized in Figure 2B. The innermost hexagon represents the parameters that result in the largest distribution ratio. Each radial section, on the other hand, displays the results obtained when only the parameters indicated are adjusted. The parameter optimization process described above represents a universal evaluation methodology that may be involved when considering different antigens enrichment and new types of ATPSs.

Next, the enrichment ratio (ER) of partitioned proteins is calculated to further evaluate the quantitative measurements of how much the target antigen will be enriched. This will provide specific fold changes in the enrichment of the target antigen. ER is defined as protein concentration in the bottom phase (C_2_) divided by the initial protein concentration (C_1_) before phase separation (Figure S2, Supporting Information). In our experiments, the initial concentration of C_1_ is obtained when diluting the known concentration of fluorescent proteins. In contrast, the enriched concentration of C_2_ is calculated using the N protein standard calibration curve in the dextran‐rich solution (Figure S2, Supporting Information). To further enhance the enrichment effect, we adjusted the PEG‐rich to dextran‐rich ratios from 1:1 to 50:1. We observed that the enrichment ratios increased with the increasing volume ratios. For instance, when the volumetric ratios increased from 1:1 to 50:1, the ER scaled up from 2.19 to 47.7 (Figure 2C). This indicates that beyond the partitioning‐induced enrichment of antigens into the bottom phase, we can further increase the enrichment by compressing the bottom phase. Similarly, by adjusting various volume ratios from 9:1 to 59:1, the viral RNA can be enriched from 11.44 to 44.51 folds, demonstrating the versatility of the phase separation‐based enrichment method (Figure 2D).

### 2.3 Flow Performance Investigation in Lateral Flow Strips

After characterizing the enrichment fold using the standard curve, we applied the enriched samples to RATs. When performing RATs, a smooth, complete liquid flow along the strip is crucial for effective signal readouts. The nitrocellulose membrane is the major component of RATs strips, upon which the enriched sample solutions are loaded.^[^ ^31^ ^]^ The capillary force drives the liquid flow within the porous membranes, which can be optimized by adjusting membrane pore size and assay viscosity.^[^ ^32^ ^]^ These issues can arise due to the smaller equivalent capillary radius and higher viscosity of assays caused by an impaired flow. It is important to note that such flow impairments can significantly hinder the ability of RATs to detect the presence of antigens accurately.^[^ ^31^ ^]^ To better understand the relationship between fluid flow and RATs performance, various models regarding the flow in porous media have been developed. For example, the solution‐wetting behaviors can be studied analytically using the Lucas‐Washburn equation (Equation 1),^[^ ^32^ ^]^ in which µ is liquid viscosity, γ is liquid surface tension, θ is the contact angle, and *r_c_* is the equivalent capillary radius. Based on this capillary model, the liquid wetted length *L* can be described as a function of time *t*.

(1) $$L\left(t\right)=\sqrt{\frac{\gamma \;\cos\theta }{2\mu }r_{c}t}$$

**Figure 3:**
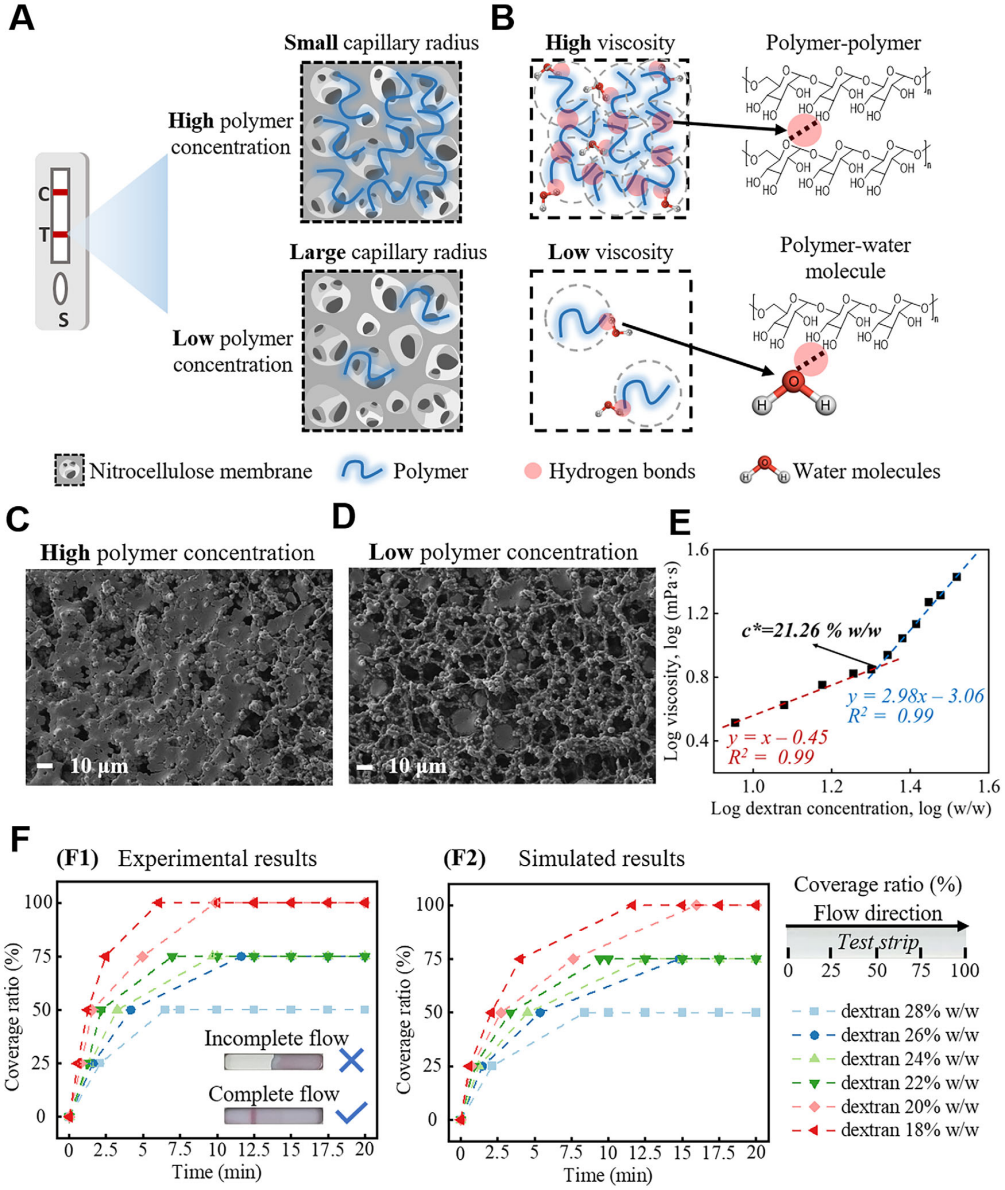
Flow performance optimization when applying the enriched antigens on RATs strips. A) Schematic illustration of nitrocellulose membranes upon loading polymer solutions with high and low concentrations. B) Schematic showing the enhanced interactions between polymers when the concentration of dextran is increased. This increase in concentration leads to a higher viscosity of the dextran solution. SEM images of the nitrocellulose membrane after loading the C) 28% wt and D) 18% dextran solutions. E) Determination of the overlap concentration (c*) by plotting the log of normalized viscosity versus the log of polymer concentrations. The linear fitting results yielded two linear regions, the intersection of which gives c*. F) Experimental (F1) and simulated (F2) results of the liquid coverage ratio, which varies as a function of time when the dextran mass fraction is increased from 18 to 28% wt. The inset images in F1 display the experimental results of both incomplete and complete flow when loading dextran solutions with low and high concentrations, respectively.

Incorporating ATPSs in conventional RATs affect the above properties, and therefore, the flow performance should be investigated accordingly. In our tests, the loading assay contains the dextran‐rich solution, where dextran polymers are the major components that interact with the nitrocellulose membrane. When the concentration of dextran increases, more of the membrane pores become blocked by evaporated polymer residues, resulting in a decreased equivalent capillary radius (**Figure** **3**A). In addition to blocking the membrane pores, dextran polymers have rich hydroxyl groups (‐OH) that can form hydrogen bonds between polymers as well as with water molecules (Figure 3B). When the concentration of dextran increases, more polymer‐polymer hydrogen bonds are formed, causing the solution to become more viscous.^[^ ^33^ ^]^ The cumulative effect of decreased membrane capillary radius and increased solution viscosity ultimately leads to a slower assay flow rate. Therefore, the influence of the dextran concentration should be precisely adjusted to ensure a smooth assay flow in lateral flow strips.

To quantitatively investigate the effect of dextran concentration on the assay flow process, we consider the membrane capillary radius and assay viscosity separately. To start, we characterize the nitrocellulose membrane pore size using solutions with increased dextran mass ratios to assess the influence of polymer deposition on membrane pores. The scanning electron microscope (SEM) images (Figure 3C,D) have confirmed that as the concentration of dextran increases, the membrane pores become increasingly blocked, resulting in a reduction in the equivalent capillary radius. Second, the viscosity and overlap concentration of dextran solutions are measured accordingly (Figure 3E; Figure S3, Supporting Information). Overlap concentration, often denoted as 𝑐∗, refers to the specific concentration of polymer chains in a solution at which they begin to overlap and entangle significantly. At this concentration, the polymer coils just start to touch each other, leading to the formation of a network structure. This transition drastically affects the solution's properties, such as viscosity and flow behavior.^[^ ^34^ ^]^ To guarantee the unimpeded flow of liquid on the test strips, we ensure the assay viscosity remains below the dextran's overlap concentration (c* = 21.26% w/w). Finally, in situ time‐lapse images and numerical simulation are used to evaluate the overall performance of the PSAP assay in RATs strips to evaluate the wetted length changes as a function of time, using different dextran concentrations. (Figure 3F). The finding indicated that higher dextran concentrations inhibit assay fluidity and result in incomplete flow in RATs. When the dextran concentration is lower than 20% w/w, assay liquids can cover the whole test strip within 15 min. These evaluations are crucial for optimizing the performance of RATs and developing reliable and efficient tests to detect infectious diseases.

### 2.4 Detection of SARS‐CoV‐2 Virus Using the PSAP Method

**Figure 4:**
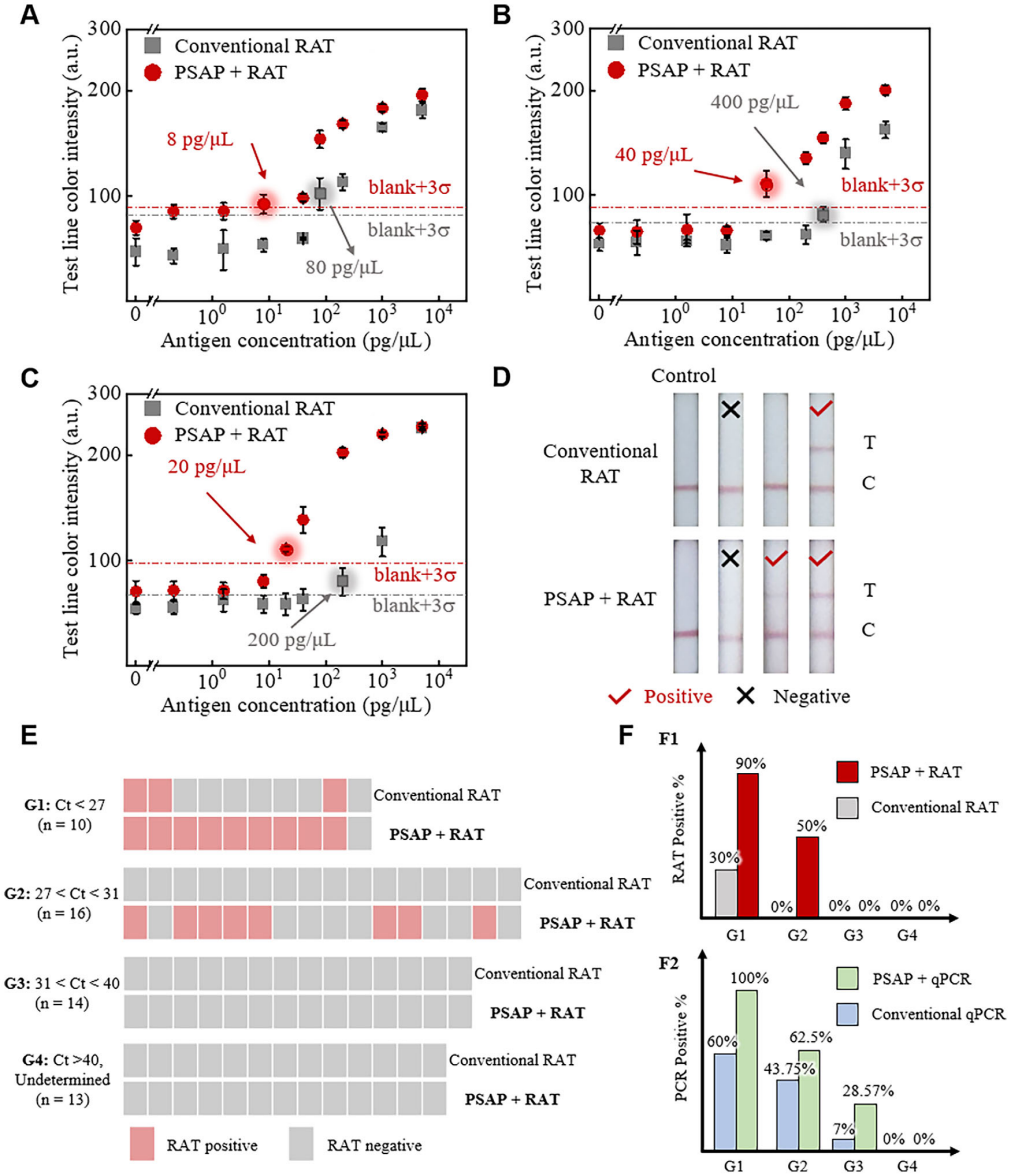
Universality and clinical validation of the PSAP method for SARS‐COV‐2 testing. A, B, C, Detection results of conventional and PSAP methods for SARS‐CoV‐2 antigens using A) Genrui brand, B) Banitore brand, and C) HighTop Brand kits. A tenfold enhancement in LOD compared to the conventional method is observed using the PSAP method. The quantitative results are plotted through image analysis using test line gray values, and the signal intensity of blank plus three times standard deviation (blank+3σ) is treated as the threshold for determining LOD.^[^ ^38^ ^]^ Clinical samples: D) Representative experimental results of RATs for samples collected from three patients and the control group without viral antigens. E) Summary of RAT results for 53 clinical trials: Group 1: undiluted samples with Ct < 27, n = 10; Group 2: Ct 27–31, n = 16; Group 3: Ct 31–40, n = 14; Group 4: Ct > 40, n = 13. F) Comparison of PSAP and conventional methods for viral antigen(F1) and RNA (F2) enrichment across clinical samples grouped by Ct values, showing enhanced detection accuracy with PSAP in low viral load specimens (G1 and G2, Ct < 31) and maintained specificity in high‐Ct (G3) and negative controls (G4). Error bars represent the standard deviation from three independent tests.

To validate the efficacy of the PSAP method, we first verify the enrichment effect of viral antigen by applying the conventional and PSAP assays in commercial RATs separately (Figure S6, Supporting Information). The volumetric ratio of PEG‐rich to dextran‐rich is kept at 9:1 for easy assay preparation. The test images of the Genrui kit are displayed in Figure S7A (Supporting Information). Image analysis results showed that commercial assays cannot generate signals when antigen concentrations are lower than 80 pg µL^−1^. In contrast, after enhancement by the PSAP method, LOD can achieve 8 pg µL^−1^, resulting in tenfold sensitivity enhancement (**Figure** **4**A). Next, to validate the generality of our PSAP method in commercial RATs, two other RATs kits, the Banitore kit, and the HighTop kit, are tested using the same experimental setups. For the Banitore kit, the LOD of single‐phase readouts is 400 pg µL^−1^, while the PSAP‐based RATs reach ≈40 pg µL^−1^ (Figure 4B; Figure S7B, Supporting Information). For the HighTop kit, the LOD of single‐phase readouts is 200 pg µL^−1^, while the PSAP‐based RATs reach ≈20 pg µL^−1^ (Figure 4C; Figure S7C, Supporting Information). The tenfold enrichment is also validated in the two kits.

After we successfully validate the efficacy of the PSAP method using reconstituted antigen dilutions, we investigate the clinical translational potential of the proposed method. Specifically, clinical samples from 53 individuals suspected of COVID‐19 are analyzed, including 40 qPCR‐positive samples (Ct values: 22.6–36.3) and 13 qPCR‐negative controls (Ct > 40). Results are compared between RATs and PCR. For antigen enrichment (Figure 4F1), original samples with Ct < 27 (Group 1, n = 10) show 30% positivity via conventional methods versus 90% with the PSAP method. In Group 2 (Ct: 27–31, n = 16), PSAP‐enriched samples yield 50% positivity, while conventional methods detect no positives. Both methods return negative results for Group 3 (Ct: 31–40, n = 14) and Group 4 (Ct > 40, n = 13). For RNA enrichment (Figure 4F2), PSAP outperforms conventional methods across all groups: Group 1 (100 vs 60%), Group 2 (62.5 vs 43.75%), and Group 3 (28.57 vs 7%), while Group 4 remains negative in both approaches. In summary, our PSAP method shows good performance both in viral antigen and RNA enrichment (Figure 4D–F, Tables S1 and S2, Supporting Information).

### 2.5 Detection of Influenza A/B Viruses and Quantitative Comparison with SARS‐CoV‐2

**Figure 5:**
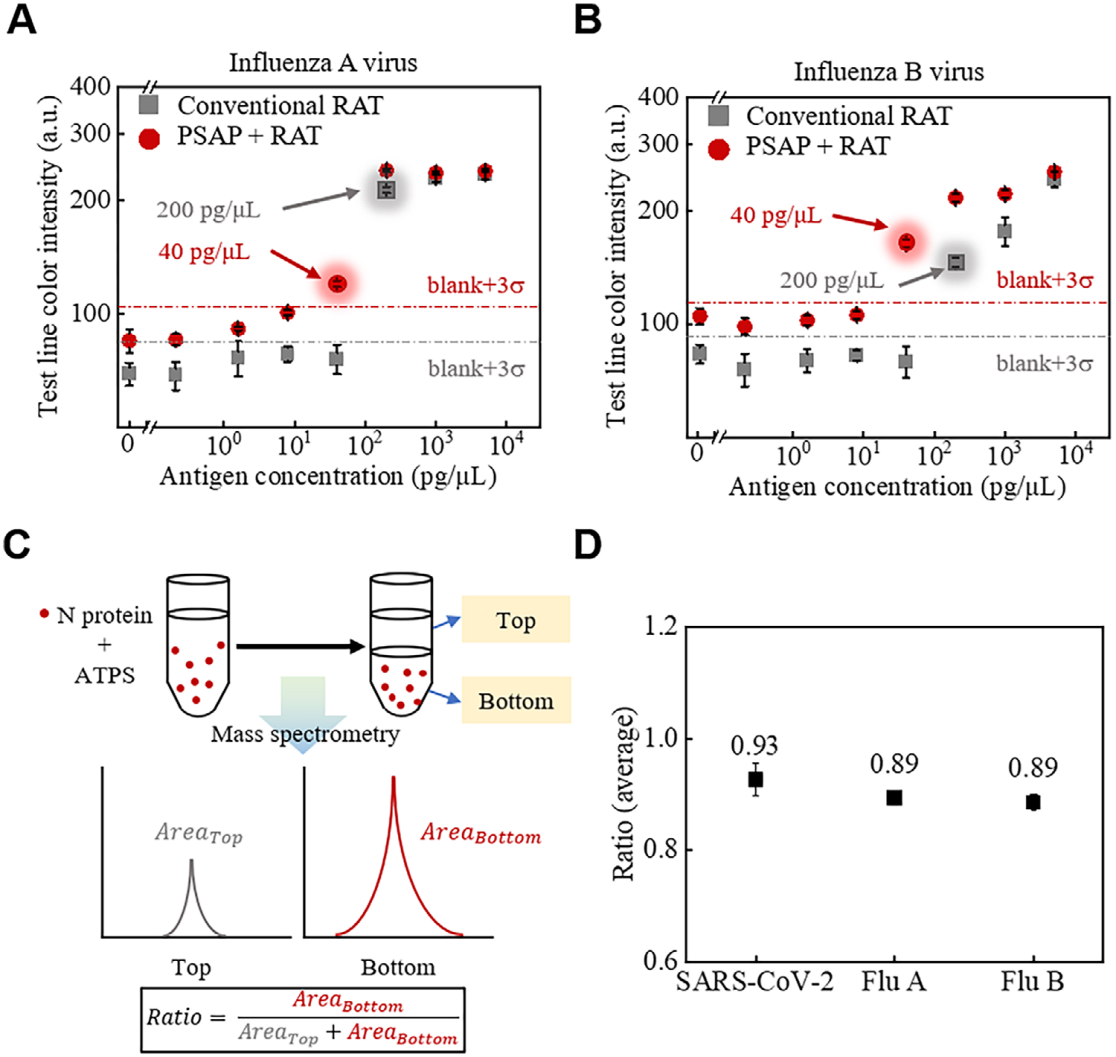
Detection of influenza A/B viruses using the PSAP method and quantitative comparison with SARS‐CoV‐2 using LC‐MS. Detection results of conventional and PSAP methods for A) Flu A and B) Flu B viruses. A fivefold enhancement in LOD compared to the conventional method is observed. C) The schematic illustrates the procedures of LC‐MS experiments. AreaTop and AreaBottom are the areas under the curve for signals in the top and bottom phases measured by LC‐MS, respectively. D) Averaged ratio of antigens for SARS‐CoV‐2, Flu A, and Flu B. Antigens from SARS‐CoV‐2 exhibited 4.5% greater phase affinity (Ratio_SARS‐CoV‐2_ = 0.93, Ratio_FluA_ = Ratio_FluB_ = 0.89). Error bars represent the standard deviation from three independent tests.

In addition, we have demonstrated the potential of applying the PSAP method to detect more types of viruses, such as the Influenza A/B viruses. (**Figure** **5**). Following the same protocols for SARS‐CoV‐2, a fivefold enrichment of LOD is observed (Figures 5A,B; S10A,B, Supporting Information). Interestingly, fewer enrichment effects are noted. To understand these phenomena, liquid chromatography‐mass spectrometry (LC‐MS) is used to investigate the partitioning of different antigens in the same ATPS (Figure 5C; Figure S12, Supporting Information). According to the amount ratio of antigens in the top and bottom phases (Figure 5D), SARS‐CoV‐2 antigens exhibit 4.5% greater phase affinity (Ratio_SARS‐CoV‐2_ = 0.93, Ratio_FluA_ = Ratio_FluB_ = 0.89). This disparity originates from distinct polymer‐antigen interfacial interactions, accounting for the different enrichment performances when testing SARS‐CoV‐2 and Influenza A/B viruses.

Inspired by these findings, we conclude that the studies presented in the current context and investigations outlined in Figure 2 primarily serve as a general framework that can be utilized in future applications. It is important to note that the enrichment procedures induced by phase separation can be generalized to enrich other biomarkers, thereby facilitating the monitoring of different diseases. Moreover, creating more versatile phase separation systems can enhance enrichment performance further. Several optimization directions warrant consideration. One potential strategy involves anchoring charged groups onto the phase separation components, which may further enhance the enrichment of oppositely charged antigens. Another approach can be the construction of multiphase separation compartments, which will selectively enrich a variety of biomarkers simultaneously. These enhancements can potentially revolutionize our method's efficiency and effectiveness.

## 3 Conclusion

In summary, this work establishes PSAP as a versatile platform for enriching trace viral biomarkers, validated through SARS‐CoV‐2 and influenza detection. The systematic optimization of ATPSs enabled precise control over biomarker partitioning. By maximizing two‐phase volume ratios, we achieve antigen and RNA enrichment factors exceeding 40‐fold. Validation across three commercial RATs kits demonstrates protocol universality, lowering tenfold detection limits to 8 pg µL^−1^ (Genrui), 20 pg µL^−1^ (HighTop), and 40 pg µL^−1^ (Banitore). The clinical translation is evidenced by analyzing 53 RT‐qPCR validated nasopharyngeal specimens (containing PCR undetectable samples as control). The PSAP method demonstrates significant performance for both viral antigens and RNA enrichment, particularly improving positivity rates in low viral load specimens (27 < Ct < 31) compared to conventional approaches while maintaining specificity in high‐Ct and negative controls. While influenza A/B testing reveals reduced enrichment efficiency, LC‐MS mechanistic studies attribute this to pathogen‐specific phase affinities (Ratio_SARS‐CoV‐2_ = 0.93, Ratio_FluA_ = Ratio_FluB_ = 0.89), highlighting opportunities for further improvements.

The PSAP has several advantages compared to other common protein precipitation methods. For instance, the acetone crash method^[^ ^7^ ^]^ is a common technique in proteomics for precipitating proteins out of a solution. While acetone precipitation effectively enriches proteins for downstream LC‐MS analysis (via pellet resuspension and enzymatic digestion), it is unsuitable for RATs due to the irreversible phase transition from liquid to solid. RATs require samples to remain in a liquid state to facilitate capillary flow and antigen‐antibody binding on the test strips. The proposed phase separation method that maintains solubility should be prioritized for point‐of‐care devices. Besides, the limited solubility of nucleic acids in acetone restricts the utility in DNA or RNA processing, thereby narrowing the range of applications. In contrast, our method successfully concentrates viral nucleic acids up to 44.51‐fold (Figure 2D), effectively addressing the limitations inherent in traditional enrichment methods.

Collectively, our phase separation‐based method offers significant potential for biomarker enrichment, particularly due to its broad compatibility and universality. The PSAP platform further employs low‐cost, commercially accessible polymers, ensuring scalability and alignment with existing industrial workflows. While further optimization is required to standardize and simplify procedural steps for non‐specialized users, its demonstrated enrichment efficacy highlights a clear pathway for advancing engineering protocols to enhance operability in diverse settings.

## 4 Experimental Section

### Ethics Statement

This study was conducted under ethical approval from the Institutional Review Board of the University of Hong Kong (UW 20–168). De‐identified nasopharyngeal samples positive for SARS‐CoV‐2 by RT‐PCR were obtained from routine public health surveillance.

### Chemicals and Reagents

PEG polymers with various molecular weights (Mw = 8,000 Da, 10,000 Da, and Mw 20,000 Da) was purchased from Sigma‐Aldrich; dextran (Mw = 10,000 Da, 70,000 Da, 500,000 Da) was purchased from Macklin; 4‐(2‐Hydroxyethyl)‐piperazine‐1‐ethanesulfonic acid with various pH values (HEPES, pH 6.5, 7.4, 9.0) and phosphate‐buffered saline (PBS, 50 mmol L^−1^, pH 7.4) was purchased from Macklin. UltraPure distilled water (DNAse, RNAse, Free) was purchased from Invitrogen. The recombinant SARS‐CoV‐2 BA.2 nucleocapsid protein with His‐tag was purchased from R&D Systems Hong Kong Limited. The influenza virus A (A/Wisconsin/588/2019) and virus B (B/Austria/1 359 417/2021) were ordered from Sino Biological. The FITC conjugation kit (Lightning‐Link (ab102884)) was purchased from Abcam. QIAamp Viral RNA Mini Kits were purchased from Qiagen. TaqMan Fast Viru 1‐step Master Mix was purchased from ThermoFisher. All the primers and probes were purchased from Integrated DNA Technologies. The EasyPep MS Sample Prep Kit (A40006, Mini MS Sample Prep Kit) was ordered from Thermo Scientific.

### ATPSs Preparation

Many polymers/salts can form ATPSs when their concentrations are sufficiently high.^[^ ^16^ ^]^ In this work, three groups of ATPSs were chosen, 1) PEG and dextran, 2) PEG and Ficoll, and 3) PEG and Sodium citrate as examples to demonstrate the general rationale when developing the ATPSs‐enhanced ultrasensitive rapid antigen tests. For each ATPSs group, a 50 g stock solution was prepared by dissolving phase‐forming materials in buffers. The resulting ATPSs mixture was centrifuged for 70 minutes at 9000 rpm and statically settled overnight. The phase‐separated upper and bottom phases were carefully separated via pipette and recombined at different upper‐to‐lower volumetric ratios in different experiments. All the working solutions were stored in a 4 °C refrigerator and used up within one week.

### Partitioning Affinities of Viral Antigen and RNA in ATPSs

To quantitatively investigate and compare antigen partitioning affinities in various ATPSs combinations, fluorescent labels were used to indicate the distribution preferences of antigens between two phases. Specifically, the N protein was labeled with fluorescein isothiocyanate (FITC) and then partitioned during the phase separation. After phase separation, the solutions were split into top and bottom phases. Then, the fluorescence intensities in the two phases were characterized using the microplate reader separately, providing information on the partitioning trend of the N protein, which was referred to as the DR.^[^ ^17^ ^]^ Similarly, to quantitatively investigate and compare partitioning affinities of viral RNA in various ATPS combinations, after the phase separation process, RT‐qPCR was used to measure the viral RNA in two phases.

### Standard Dilutions of Reconstituted SARS‐CoV‐2 N Protein

N protein stock solutions were reconstituted in the experiments following the product manual. Specifically, 100 µL of PBS (pH 7.4) was added to 100 µg of lyophilized protein powder to achieve a protein stock concentration of 1 µg µL^−1^. The reconstituted protein was stored in a 4° refrigerator and tested within one month. In the subsequent experiments, N protein dilutions were prepared using various buffers and ATPSs solutions according to different parameters under investigation.

### Calibration Curves of Fluorescent N Protein

To measure the calibration curve of N protein, the reconstituted N proteins were labeled using FITC, following the standard protocol of the labeling kit (Lightning‐Link, ab102884, Abcam). The N protein solutions with various concentrations were prepared by serially diluting the stock N protein in the extracted dextran‐rich phase. All the N proteins were relocated in 96‐well plates and characterized by Spectra Max iD3 multi‐mode microplate reader (Molecular Devices, CA).

### Quantitative Characterization of Fluorescent and Untagged Antigens

To compare the effects of FITC on the phase preference of analytes, the LC‐MS experiments were utilized to quantitatively measure the fluorescent and untagged antigens in different aqueous phases. The protocols of LC‐MS were followed in the literature.^[^ ^7^ ^]^ All the results and experiments can be found in the supporting materials.

### RATs Validation for SARS‐CoV‐2 and Influenza A/B Antigens

To evaluate the LOD enhancement enabled by the PSAP method in conventional RATs and to validate the general utility of the method, conventional and PSAP assays were conducted and compared in three different RATs kits on the market. Reconstituted SARS‐CoV‐2 antigen dilutions were prepared for control experiments using various concentrations (from 0.32 to 5000 pg µL^−1^) and 10 mM HEPES buffer solution. For PSAP groups, the antigens were prepared using recombined ATPSs mixtures with a PEG‐rich to dextran‐rich volumetric ratio of nine. The starting concentrations of each dilution were the same as their single‐phase counterparts. The RATs for SARS‐CoV‐2 N protein dilutions were performed by pipetting a 100‐µL aliquoted N protein solution into the sample loading area of the test strips. The conventional and the ATPSs‐enriched samples were prepared and tested within one day. Following the same steps for the SARS‐CoV‐2 virus, the antigens of the Influenza A/B virus were serially diluted using single‐phase buffers and ATPSs separately. The RATs for Influenza A/B virus antigens were performed by pipetting a 100‐µL aliquoted N protein solution into the sample loading area of the test strips. The single‐phase and the ATPSs‐enriched sample solutions were prepared and tested within one day.

### RATs for SARS‐CoV‐2 Clinical Specimens

Upon receiving the clinical samples from collaborators, those viral samples were aliquoted and stored in a ‐80 °C freezer. Before testing the viral samples using commercial rapid antigen test kits, the working assays were prepared by constructing conventional (single‐phase) buffers and PSAP solutions (two‐phase) separately. A 500‐fold dilution step was involved during the assay preparation process, leading to new Ct values in conventional and PSAP groups (Figure S9, Supporting Information). For each group, first, 405 µL lysis buffer was added to 45 µL vial sample, and the incubation time was 10 min; second, 20 µL lysed virus was added to conventional buffer and PSAP solution separately, and the two mixture were mixed completely by shaking; thirdly, the two solutions were put into centrifuge to spin 3 min at 5000 rpm; finally, the bottom phase of PSAP group was extracted and applied to the RATs, while the conventional buffer was applied to RATs directly as comparison. After 15 minutes, the RATs results were obtained. In total, the RATs tests were completed within 30 minutes. The RATs for SARS‐CoV‐2 clinical samples were performed by pipetting 130‐µL aliquoted working assays to the sample loading area of the test strips. The single‐phase and the ATPSs‐enriched samples were prepared and tested within one day.

### RT‐qPCR Validation of Enriched SARS‐CoV‐2 Viruses

The Viral RNA purification kit (QIAamp Viral RNA Mini Kit, Qiagen) was used for RNA extraction, as instructed by the manufacturer. RNA was extracted from 70 µL of the samples and eluted in 60 µL elution buffer. A 20 µL RT‐qPCR assay contained a master reaction mixture (TaqMan Fast Virus 1‐step Master Mix, Thermo Fisher), N‐gene primers, a probe, and a 4 µL RNA sample. RT‐qPCR reactions were performed by a thermal cycler, with the following conditions: reverse transcription at 50 °C for 5 minutes, inactivation of reverse transcriptase at 95 °C for 20 s, and 45 cycles of PCR amplification (Denaturing at 95 °C for 5 s; Annealing/Extending at 58 °C for 30 s).

### RATs Colorimetric Image Process and Analysis

Images of RATs were captured using cell phones under fixed light source conditions. Then, the color images were processed by ImageJ software (NIH)^[^ ^35^ ^]^ to extract the grey intensity of “test” and “control” lines: First, the pristine images were converted into 8‐bit type; second, the image color was inverted; finally, use the line measurement tool to obtain the grey values of “test” and “control” lines. To mitigate the intensity variations due to photographing, the test line signals were normalized by the averaged edge intensity measured for negative controls, assuming the negative control should have the same signal intensity between independent tests. The sample concentrations can be reflected by the signal intensities following these processing steps. Then, the LOD information can be obtained after plotting the test line signal intensities versus the antigen concentrations.

### Scanning Electron Microscope (SEM) Experiments

The morphology of nitrocellulose membranes was characterized by using a Zeiss scanning electron microscope (Gemini SEM 300) at an accelerating voltage of 10 kV. The samples were prepared by loading dextran solutions on nitrocellulose membranes with increased concentrations and air‐drying (28, 26, 24, 22, 20, and 18 wt%).

### Numerical Simulation of the Liquid Flow in Test Strips

The Computational Fluid Dynamics (CFD) simulation of assay flow in the RATs strips was conducted using the Fluid Flow Module in COMSOL Multiphysics software (version No. 6.2). It has been suggested that a 2D configuration was sufficiently accurate for the simulation of the lateral flow assay.^[^ ^36^ ^]^ Accordingly, a 2D model was built of the test strip using the measured geometry parameters (Figure S4 and Table S5, Supporting Information). Subsequently, the computational mesh was constructed using the built‐in mesh setting function in COMSOL. The Sequence Type and Element size of the mesh file were set to “Physics‐controlled mesh” and “Extremely fine”, respectively. The unsaturated liquid flow in the porous membranes can be described by the Richard's equation.^[^ ^37^ ^]^ Under varying dextran concentrations, the properties of the liquid, including viscosity, surface tension coefficient, surface contact angle, and density, will differ (Table S6, Supporting Information). Detailed parameters about the CFD modeling can be found in the supplementary materials (Table S7, Supporting Information). The simulation time ranges from 0 to 1200 s. The cessation of flow was defined when the flow front had not attained a predetermined percentage of the test membrane, but the time had surpassed 1200 s. According to the simulation results, the time was recorded when the solutions reached different coverage ratios, namely 25, 50, 75, and 100% of the test membranes. Then, the time points as the x‐axis and coverage ratios were plotted as the y‐axis (Figure 3F2; Figure S5 and Table S8, Supporting Information).

### Statistical Analysis

All the experiments in this work were repeated at least three times. The average and standard errors were calculated using the STDEV function in EXCEL. To define the LOD of commercial RATs and phase separation‐assisted RATs, the LOD threshold value equal to the color intensity of the blank group was set, adding three times the standard deviations (blank + 3σ).^[^ ^38^ ^]^ Due to different background colorations, independent background thresholds were used to calculate the limit of detection of conventional and PSAP groups. Besides, the signal‐to‐noise ratio (SNR) was calculated by dividing the extracted signal by the background (Figures S8 and S11, Supporting Information).

(2) $$Signal-to-noise\;ratio\;\left(SNR\right)=\frac{Signal}{Noise}$$

## Conflict of Interest

Ho Cheung Shum holds shares in, or acts as advisor of MicroDiagnostics Limited, PharmaEase Tech Limited, Upgrade Biopolymers Limited, Monexus Innovation Limited, Multera Inc, EN Technology Limited, and Capsum, and he is the Founding Centre Director & Co‐Director of Advanced Biomedical Instrumentation Centre Limited. The works in this paper are, however, not directly related to the works of these entities, as far as we know.

## Supporting information



Supporting Information

## Data Availability

The data that support the findings of this study are available from the corresponding author upon reasonable request.
