# Supplementary material for: A Phase Separation‐Assisted Pre‐Enrichment Method for Ultrasensitive Respiratory Virus Detection
Source: Adv Sci (Weinh). 2025 Jun 27;12(36):e06578. doi: 10.1002/advs.202506578 (PMC12463056; doi:10.1002/advs.202506578)
Supplement: Supplementary file 1 — Supporting Information [file ADVS-12-e06578-s001.docx]

**Supplementary information**

**A PHASE SEPARATION-ASSISTED PRE-ENRICHMENT METHOD FOR ULTRASENSITIVE RESPIRATORY VIRUS DETECTION**

Yang Cao^1, 2^, Pui Ngan Lau^3^, Alex W.H. Chin^3^, Zhuolin He^1, 6^, Christina C. K. Au Yeung^1, 2^, Kehao Zeng^1^, Haisong Lin^1,2,7,8, *^, Leo L. M. Poon^3,4,5,*^, and Ho Cheung Shum^1, 2, 9, 10*^

1 Department of Mechanical Engineering, The University of Hong Kong, Pokfulam Road, Hong Kong SAR, 000000, China

2 Advanced Biomedical Instrumentation Centre, Hong Kong Science Park, Shatin, New Territories, Hong Kong SAR, 000000, China

3 Division of Public Health Laboratory Sciences, School of Public Health, Li Ka Shing Faculty of Medicine, The University of Hong Kong, Hong Kong SAR, 000000, China.

4 HKU-Pasteur Research Pole, School of Public Health, Li Ka Shing Faculty of Medicine, The University of Hong Kong, Hong Kong SAR, 000000, China.

5 Hong Kong Jockey Club Global Health Institute, Li Ka Shing Faculty of Medicine, The University of Hong Kong, Hong Kong SAR, 000000, China

6 Weiyang College, Tsinghua University, Beijing, 100084, China.

7 School of Engineering, Westlake University, Hangzhou, 310030, China.

8 Research Center for Industries of the Future, Westlake University, Hangzhou, 310030, China.

9 Department of Biomedical Engineering, City University of Hong Kong, Hong Kong SAR, 000000, China

10 Department of Chemistry, City University of Hong Kong, Hong Kong SAR, 000000, China

* Corresponding email: linhaisong@westlake.edu.cn, [llmpoon@hku.hk](mailto:llmpoon@hku.hk), ashum@cityu.edu.hk

**Liquid chromatography-mass spectrometry (LC-MS)**

Sample preparation

The reconstituted antigen solutions (around 60 μL) were transferred to a microcentrifuge tube and adjusted final volume of 100 μL with lysis solution. Then the reduction solution (50 μL), alkylation solution (50 μL) were added in sequence and gently mixed. Then the samples were incubated at 95°C using a heat block for 10 minutes to reduce and alkylate the proteins. After incubation, 50 μL reconstituted enzyme solution (add 500 μL enzyme reconstitution solution to 1 vial of Trypsin/Lys-C Protease Mix) was added to cooled protein samples and incubated at 37°C for two hours. After incubation, 50 µL of the digestion stop solution was added to the sample and gently mixed. Then, the digested samples were cleaned up using specialized peptide clean-up columns, following the kit protocols. Finally, dried the peptide samples using a vacuum centrifuge (Centrifugal Vacuum Concentrator, HyperVAC-LITE) for 12 hours. The dried samples were stored in a 4°C refrigerator and resuspended in 50 μL of 0.1% formic acid in water for LC-MS analysis.

LC-MS method

LC-MS was performed using the Sciex TripleTOF 6600 coupled with Exion UHPLC. The column was Waters ACQUITY UPLC BEH C18 1.7µm 2.1x50mm, the mobile phase A was 0.1% formic acid in H_2_O, the mobile phase B was 0.1% formic acid in ACN. The injected volume was 2 μL, and the column temperature was 45°C. The total run time was 15 minutes, with a flow rate of 0.4 mL/min. The liquid elution profile was summarized in Table S2. The pressure of the curtain gas and ion source gas was 25 psi and 60 psi, respectively. The operating temperature was 600°C. The ion spray voltage floating was 5500V. The MS1 spectra were collected from 300-1050 m/z for 100 ms. Parent ions of corresponding peptides with strong signal intensity were selected for fragmentation (Table S3). MS2 spectra of the selected parent ions were collected from 300-1000m/z for 250 ms. Peak areas of product ions, which reflect the protein level, were calculated using the MultiQuant software (Ver 1.7).

As illustrated in Figure S12 (A), we calculate the ratio by dividing the *Area_Bottom_* by the sum of *Area_Top_* and *Area_Bottom_*. This method determines the fraction of proteins partitioned into the bottom phase. In the case of the SARS-CoV-2 antigen, we utilize three peptide fragments in the mass spectrometry experiment. These fragments possess mass/charge values of 471.8, 416.2, and 687.4, respectively, as indicated in Figure S12 (B) and Table S3. The results indicate that both untagged and FITC-labelled antigens migrate into the bottom phase (dextran-rich phase) after phase separation. The untagged antigen ratio is marginally less than its FITC-labelled counterpart, a discrepancy likely due to the hydrophobicity induced by FITC.

Regarding the antigen of Flu A, we identify two peptide fragments with mass/charge values of 662.8 and 512.3. The protein partitioning results also follow the same trend with SARS-CoV-2, that both untagged and labelled antigen prefer the dextran-rich phase. Similarly, the FITC label appears to enhance the partitioning process (see Figure S12 (C), Table S3).

In the case of the antigen of Flu B, we use three peptide fragments with mass/charge ratios of 480.3, 498.8, and 522.3. Our results suggest that both untagged and labelled antigens are partitioned into the dextran-rich phase, with FITC having no discernible impact on the partitioning results (see Figure S12 (D) and Table S3).

Upon comparison of the ratios among SARS-CoV-2, Flu A, and Flu B, it becomes apparent that the current ATPS composition is most effective for partitioning the SARS-CoV-2 antigen. This is due to the larger ratio observed for SARS-CoV-2 compared to Flu A and Flu B. These results also explain the varying sensitivity observed in rapid antigen tests.

**CFD modelling of the liquid flow in test strips**

Richard’s equation can be used to depict the unsaturated liquid flow in the porous material[1].

$$\rho(\frac{C_{m}}{\rho g}+S_{e}S_{p})\frac{\partial p}{\partial t}+\nabla\cdot\left( \rho\boldsymbol{u} \right)=0$$

$$\boldsymbol{u}=-\frac{\kappa}{\mu}\nabla(p+\rho gz)$$

$\kappa$, the permeability of the fluid, can be interpreted by the following empirical formula:

$$\kappa=\kappa_{s}\kappa_{r}(Se)$$

$\kappa_{s}$ gives the hydraulic (absolute) permeability (i.e., permeability when porous material is saturated with the fluid), and $\kappa_{r}$ denotes the relative permeability. Here, the Brooks and Corey retention model is used, which is both supported by Richard’s equation branch and confirmed by the case in the COMSOL Blog[2-3]. Under varying dextran concentrations, the properties of the liquid, including viscosity, surface tension coefficient, surface contact angle, and density, will differ. The theoretical and physical parameters used in the modelling are shown in Tables S4-S6.

The boundary condition of the sample hole is set to be a piecewise function:

$$H_{p}=\left\{ \begin{matrix} \frac{p_{0}}{\rho g}, t\in\left[ 0 , 0.5s \right]\cup\left[ T+0.5s , +\infty\right] \\ H_{w}\frac{T-t}{T},t\in\left[ 0.5s , T+0.5s \right] \end{matrix} \right.$$

$$T=120s\cdot\frac{\mu}{\mu_{0}}$$

in which, $p_{ec}=\frac{4\gamma\cos\Theta}{d_{po}}$, $p_{0}=-p_{ec}\cdot{Se}_{0}^{-\frac{1}{n}}$, according to the Brooks and Corey retention model[2]. The formula of *T* is defined due to the fluid velocity is inversely proportional to $\mu$. Under the same cross section, the flow rate is also inversely proportional to $\mu$, then the time to completely absorb the sample fluid is proportional to $\mu$. Hw, the water head, equals to 2 mm, and the ${Se}_{0}$, the initial residual wetting phase saturation, equals 0.025. We considered the volume that $Sⅇ=\frac{\theta-\theta_{r}}{\theta_{s}-\theta_{r}}\text{>0.075}$, which is 5 times more than the initial condition, as covered by the sample fluid, and checked the time when the fluid has reached and passed 25%, 50%, 75%, and 100% of the 20mm test membrane part.

**Supplementary Figures**


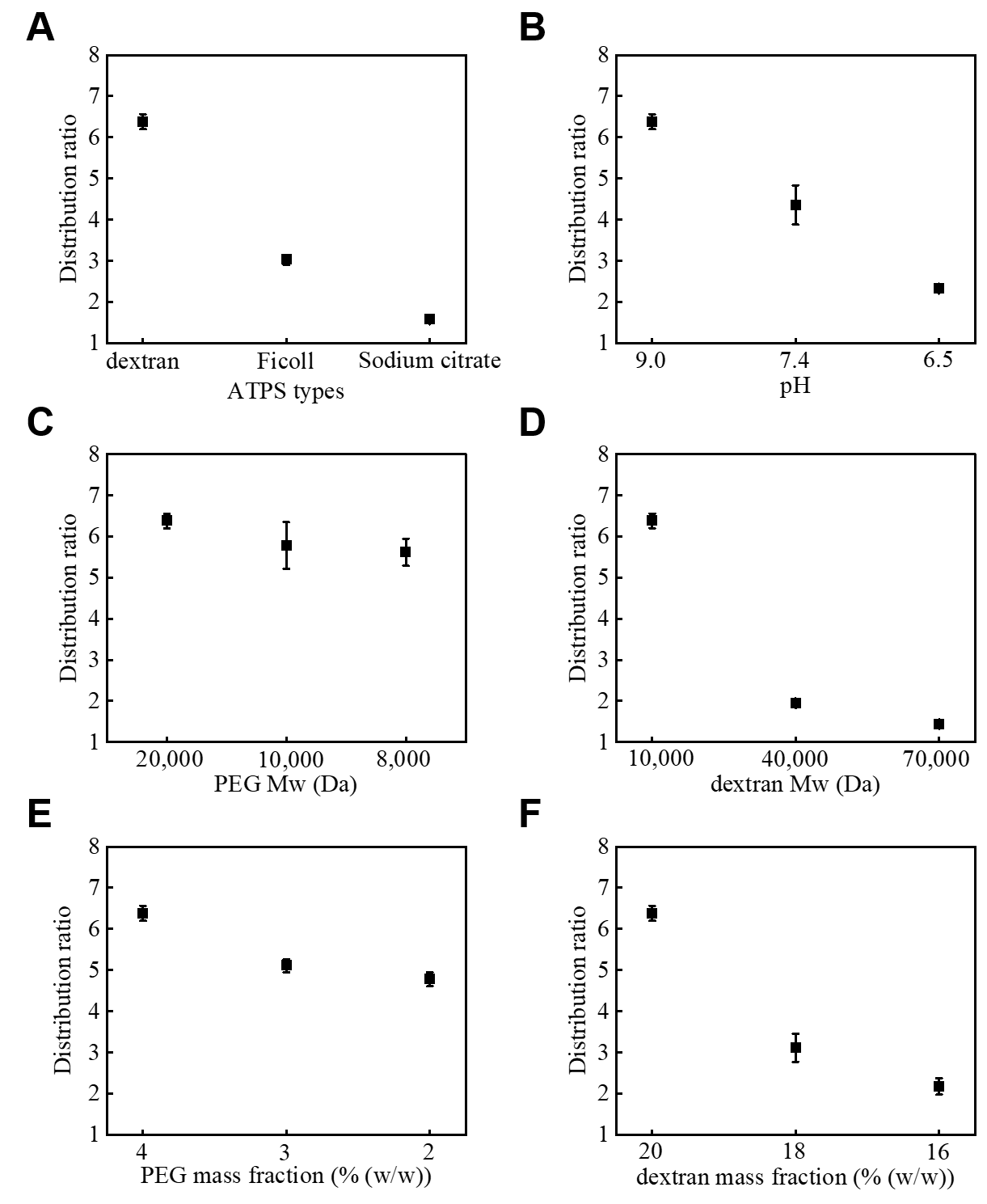


**Figure S1** Distribution ratios of SARS-CoV-2 nucleocapsid protein in ATPSs by adjusting composition parameters including (**A**) ATPS types, (**B**) buffer pH values, (**C**) PEG molecular weights, (**D**) dextran molecular weights, (**E**) PEG mass fractions, and (**F**) dextran mass fractions, respectively. Standard error bars represent triplicate experiments.


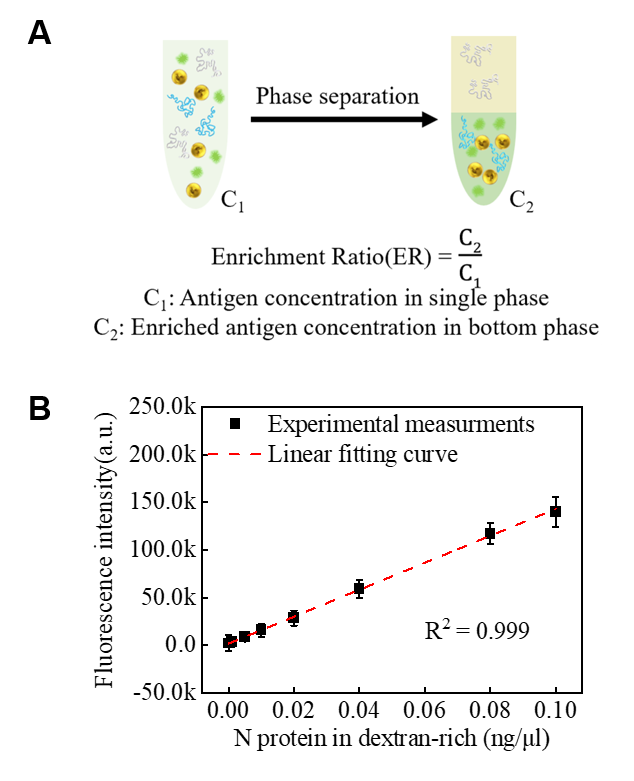


**Figure S2** (**A**) Illustration and definition of the antigen enrichment ratio (ER); (**B**) The calibration curve of antigen dilutions in the dextran-rich phase. The concentrations of antigens are: 0 ng/μL, 0.001 ng/μL, 0.005 ng/μL, 0.01 ng/μL, 0.02 ng/μL, 0.04 ng/μL, 0.08 ng/μL and 0.1 ng/μL.


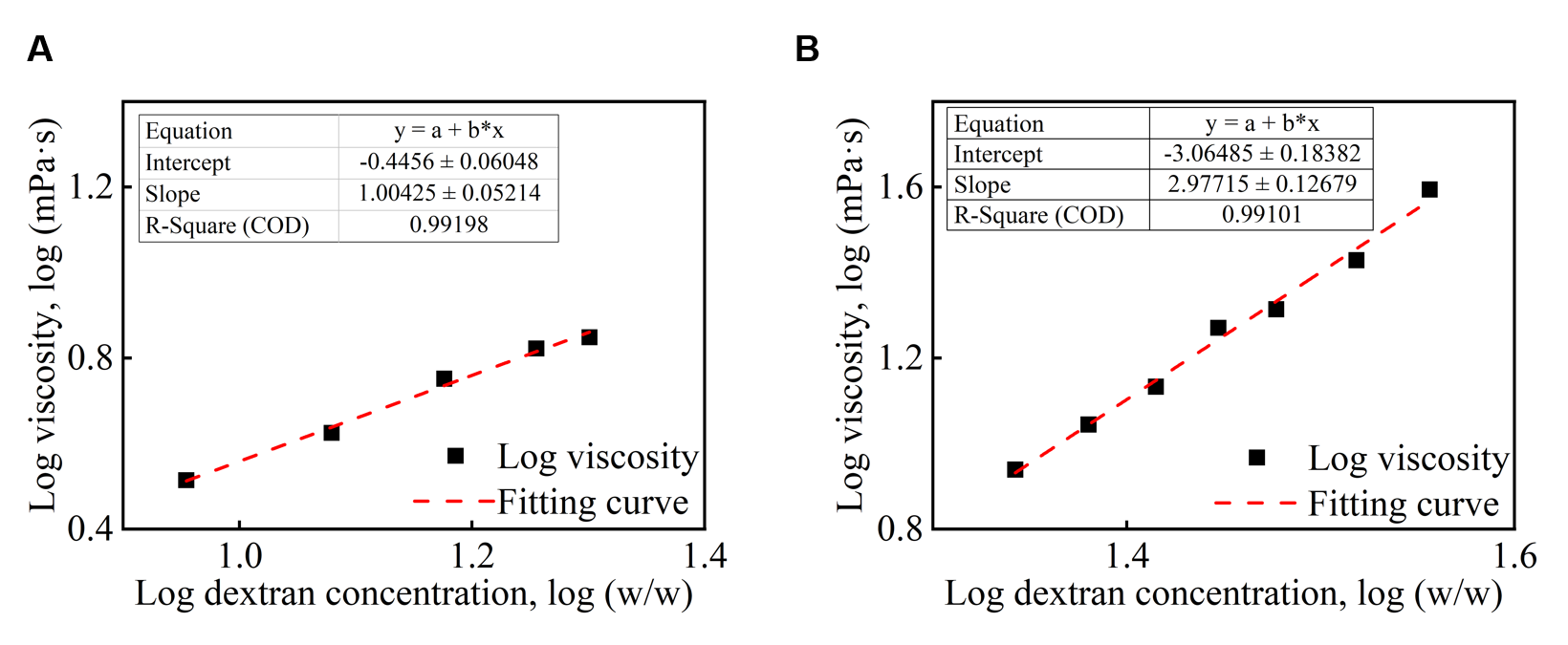


**Figure S3:** Fitting curves of the logarithm of normalized viscosity versus the logarithm of polymer concentrations. (**A**) represents concentrations smaller than the overlap concentration c*, and (**B**) represents concentrations larger than the overlap concentration c*.


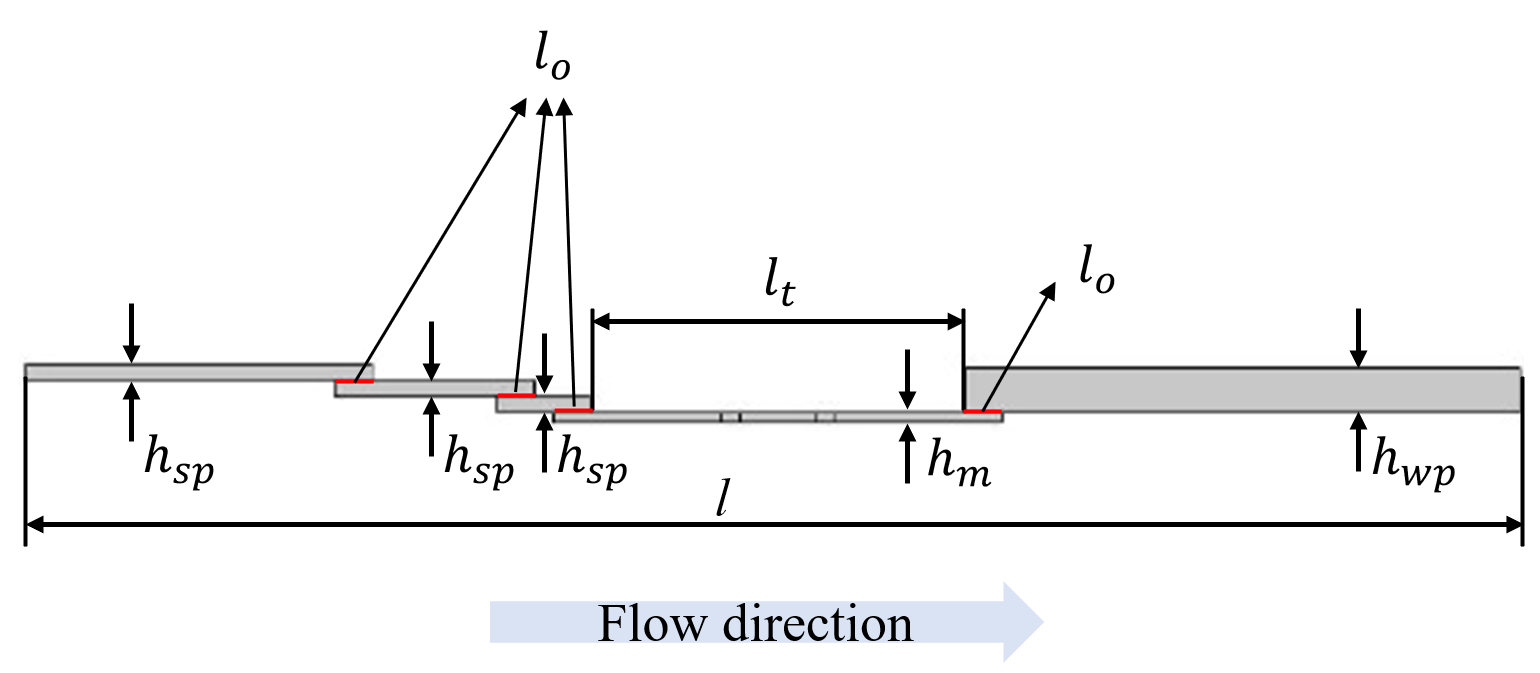


**Figure S4** Geometry parameters of the test strip for 2D simulation.


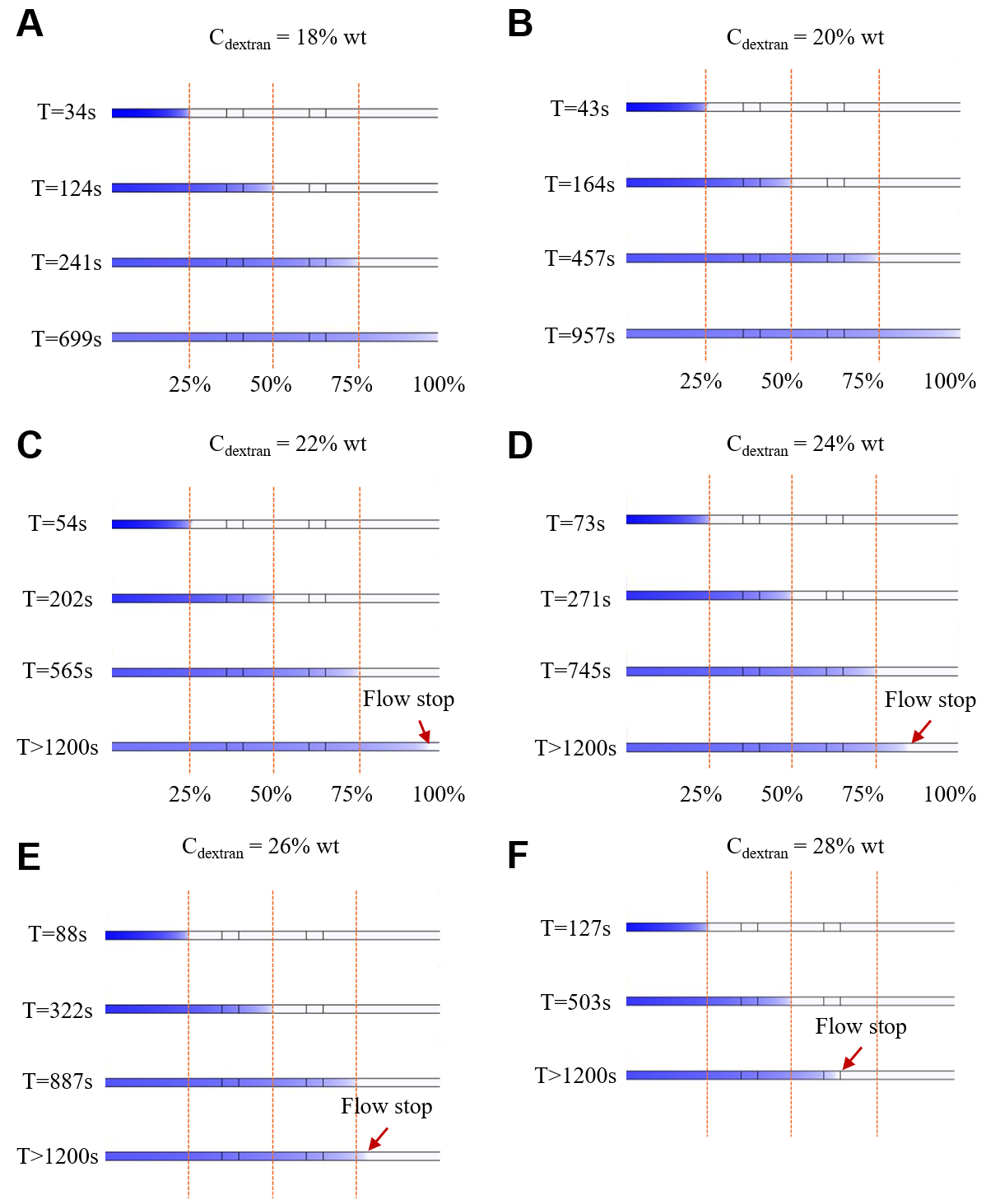


**Figure S5** Simulated results of liquid flow inside test membranes with increasing dextran concentrations: (**A**) 18% wt, (**B**) 20% wt, (**C**) 22% wt, (**D**) 24% wt, (**E**) 26% wt, and (**F**) 28% wt.


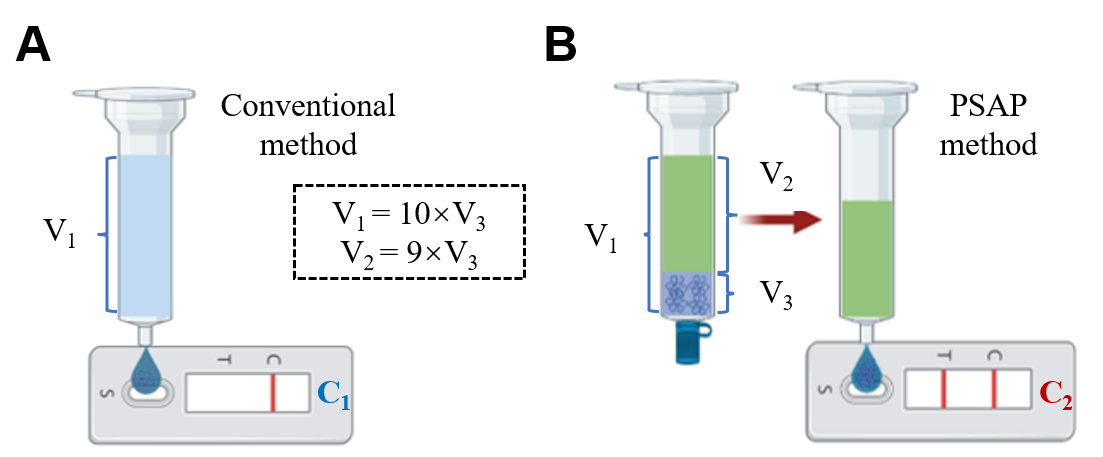


**Figure S6** Schematic illustrates implementation steps for RATs using (**A**) conventional and (**B**) PSAP methods.


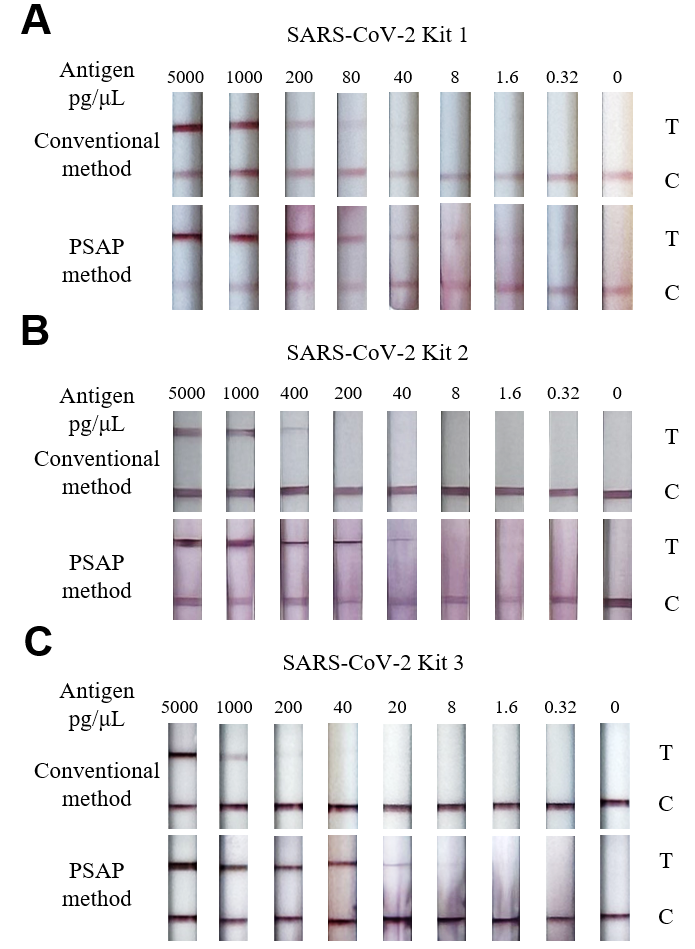


**Figure S7** RAT results of conventional and PSAP methods for SARS-CoV-2 antigens using (**A**) Genrui brand, (**B**) Banitore brand, and (**C**) HighTop Brand kits.


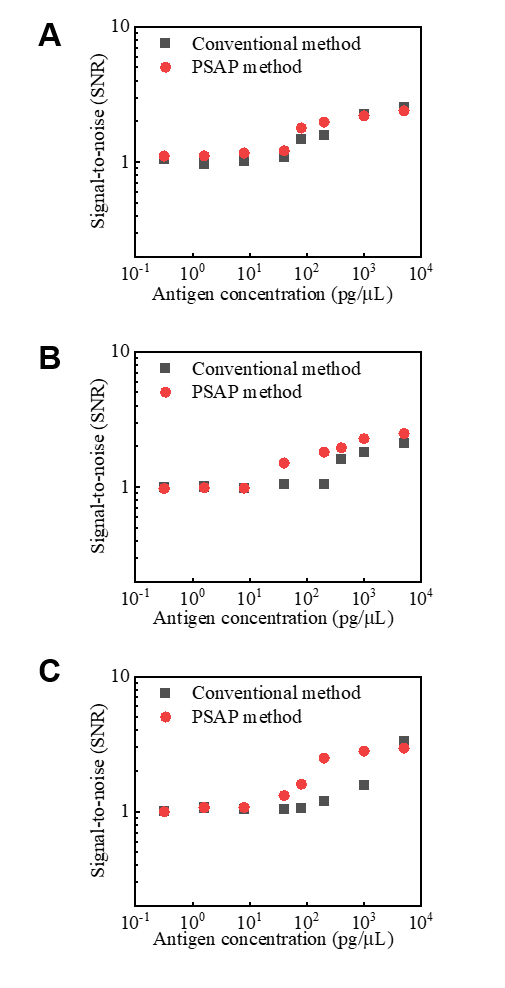


**Figure S8** Signal-to-noise ratio (SNR) of RAT results using (**A**) Genrui brand, (**B**) Banitore brand, and (**C**) HighTop brand kits.


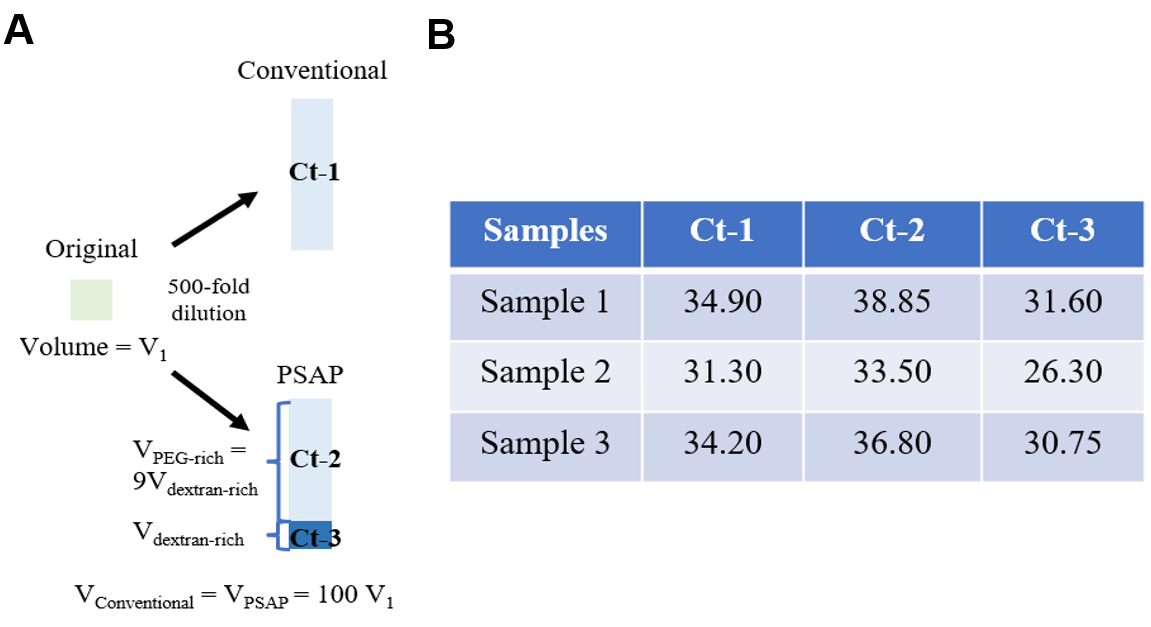


**Figure S9** Clinical sample tests using PSAP and conventional methods. **(A)** The schematic illustrates the dilution process when reconstituting the conventional (single-phase) and PSAP (two-phase) samples from clinical samples. **(B)** RT-qPCR results in conventional assay (Ct-1), PEG-rich phase (Ct-2), and dextran-rich phase (Ct-3).


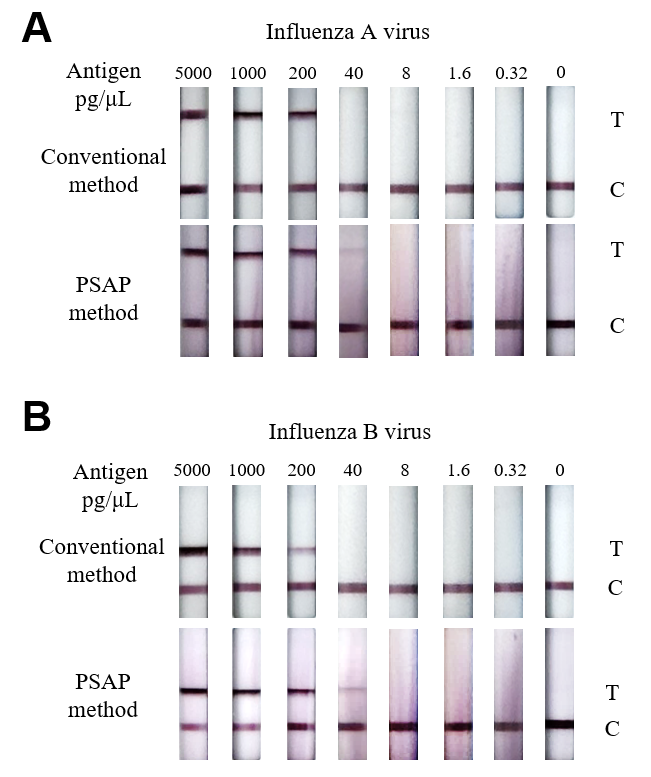


**Figure S10** RAT results of conventional and PSAP methods for detecting reconstituted N proteins of (**A**) influenza A virus and (**B**) influenza B virus using a commercial RAT kit.


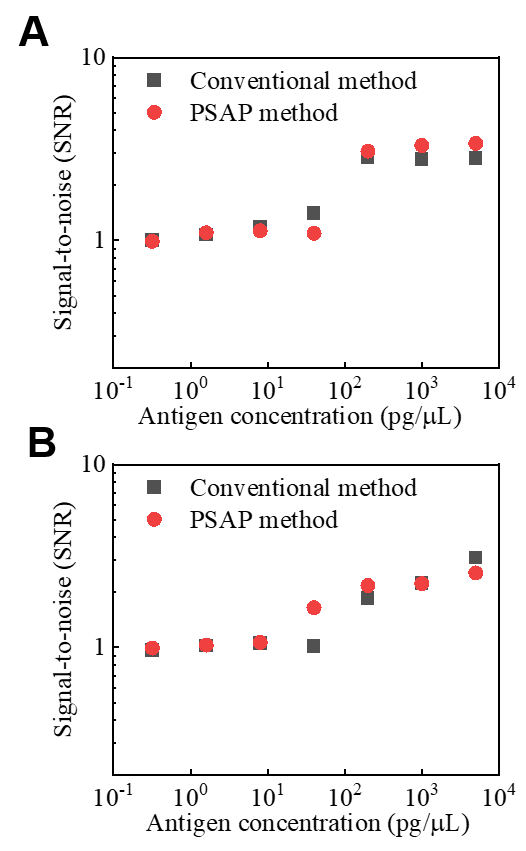


**Figure S11** SNR of RAT results when testing (**A**) Influenza A and (**B**) Influenza B viruses.


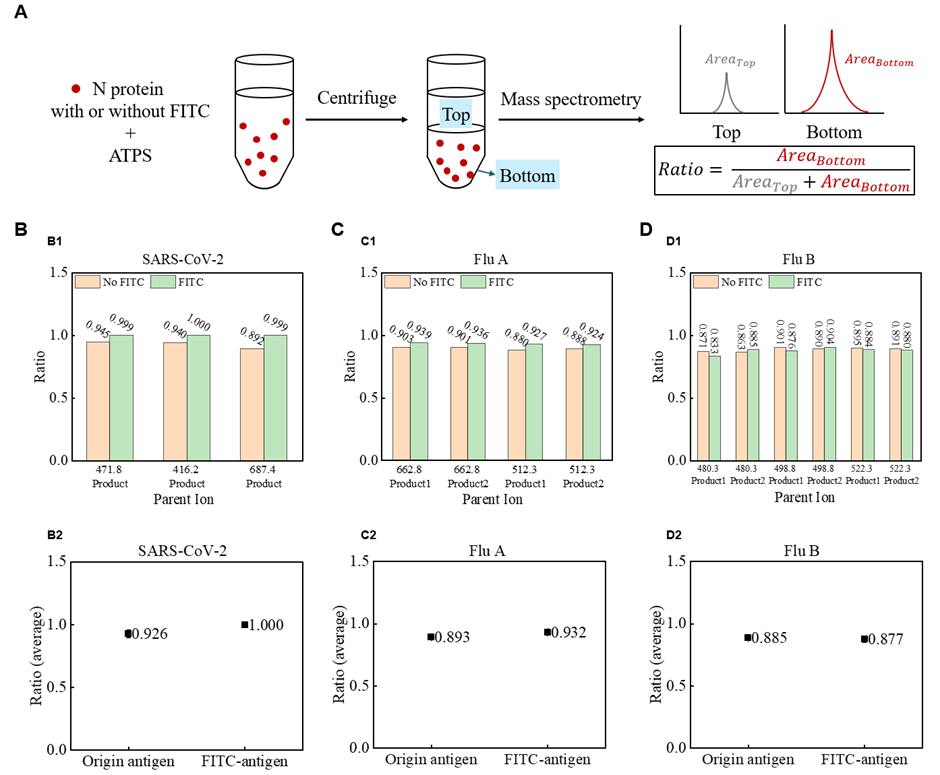


**Figure S12** Partitioning of origin and FITC-conjugated antigens in ATPS measured by LC-MS. (**A**) The schematic illustrates the procedures of LC-MS experiments. *Area_Top_* and *Area_Bottom_* are the areas under the curve for the top and bottom phases measured using LC-MS, respectively. (**B**) Partitioning ratio of antigen for (**B**) SARS-CoV-2, (**C**) Flu A, and (**D**) Flu B. **B1**, **C1**, **D1**: peptide fragments of various mass/charge values; **B2**, **C2**, **D2**: averaged ratio of all peptide fragments used.

**Supplementary Tables**

**Table S1** Summary of the Ct values and RATs results for clinical samples analysed by PSAP and conventional methods, with samples grouped by ascending Ct values.

| **Sample No.** | **Count of original samples** | **Ct of**  **single-phase**  **(500-fold dilution)** | **Conventional RAT** | **Ct of**  **Dextran-rich**  **(50-fold dilution)** | **PSAP-RAT** |
| --- | --- | --- | --- | --- | --- |
| **Group 1: Original Ct < 27, n = 10** | | | | | |
| 1 | 22.6 | 31.3 | 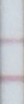 | 26.3 | 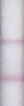 |
| 2 | 23 | 31.9 | 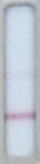 | 27.3 | 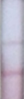 |
| 3 | 23.79 | 37.38 | 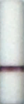 | 32.23 | 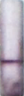 |
| 4 | 24.44 | 40.41 | 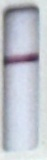 | 38.17 | 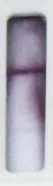 |
| 5 | 24.47 | 33 | 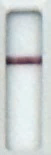 | 29.46 | 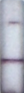 |
| 6 | 24.67 | 43.41 | 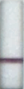 | 39.16 | 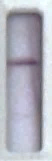 |
| 7 | 25.14 | 40.81 | 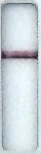 | 32.93 | 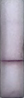 |
| 8 | 25.4 | 36.1 | 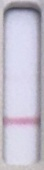 | 29.5 | 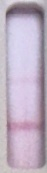 |
| 9 | 25.6 | 32.0 | 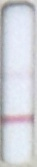 | 26.7 | 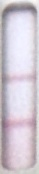 |
| 10 | 25.82 | Undetermined | 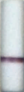 | 39.68 | 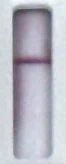 |
| **Group 2: 27 < Original Ct < 31, n = 16** | | | | | |
| 11 | 27.3 | 34.2 | 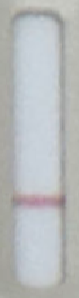 | 30.75 | 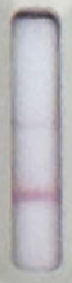 |
| 12 | 27.41 | Undetermined | 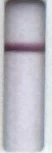 | Undetermined | 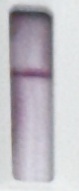 |
| 13 | 27.53 | 34.77 | 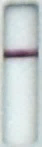 | 29.84 | 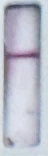 |
| 14 | 27.56 | 40.49 | 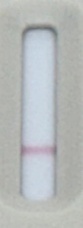 | 31.78 | 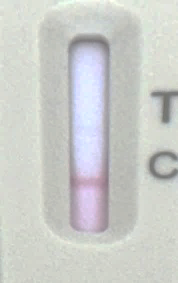 |
| 15 | 27.73 | 38.01 | 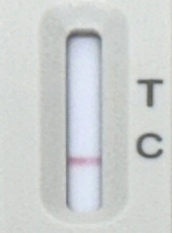 | 30.95 | 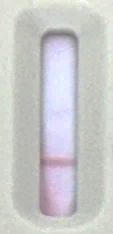 |
| 16 | 28.01 | 38.68 | 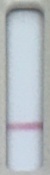 | 32.12 | 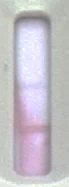 |
| 17 | 28.07 | Undetermined | 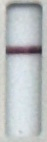 | 37.49 | 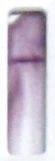 |
| 18 | 28.19 | Undetermined | 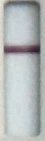 | Undetermined | 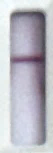 |
| 19 | 28.38 | Undetermined | 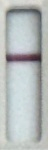 | 44.68 | 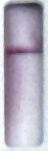 |
| 20 | 28.42 | Undetermined | 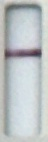 | Undetermined | 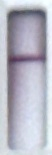 |
| 21 | 28.8 | 38.4 | 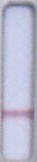 | 34.3 | 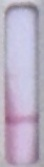 |
| 22 | 28.81 | 35.88 | 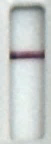 | 31.11 | 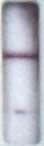 |
| 23 | 29.41 | 41.19 | 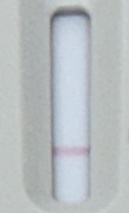 | 36.05 | 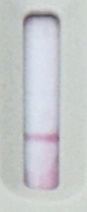 |
| 24 | 30.21 | Undetermined | 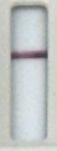 | Undetermined | 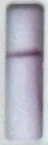 |
| 25 | 30.41 | 38.91 | 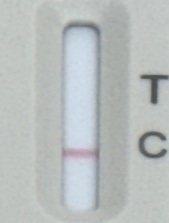 | 33.4 | 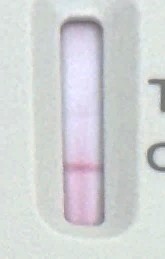 |
| 26 | 30.56 | Undetermined | 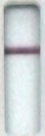 | Undetermined | 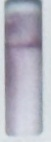 |
| **Group 3: 31 < Original Ct < 40, n = 14** | | | | | |
| 27 | 31.01 | Undetermined | 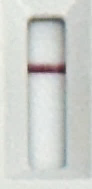 | Undetermined | 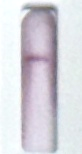 |
| 28 | 31.06 | 43.78 | 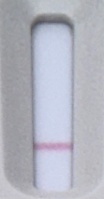 | 33.56 | 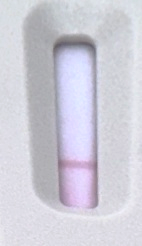 |
| 29 | 31.15 | Undetermined | 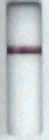 | Undetermined | 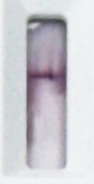 |
| 30 | 31.32 | Undetermined | 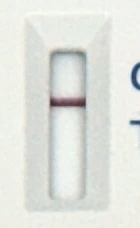 | Undetermined | 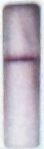 |
| 31 | 31.35 | Undetermined | 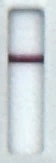 | Undetermined | 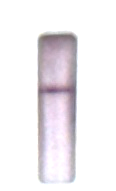 |
| 32 | 31.68 | Undetermined | 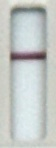 | Undetermined | 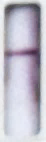 |
| 33 | 32.01 | Undetermined | 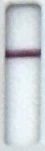 | Undetermined | 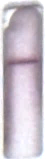 |
| 34 | 32.72 | 39.43 | 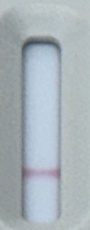 | 34.69 | 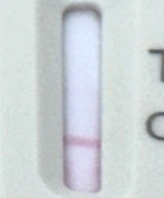 |
| 35 | 33.06 | Undetermined | 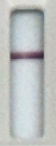 | 42.19 | 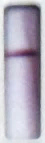 |
| 36 | 33.26 | Undetermined | 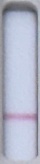 | 35.28 | 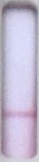 |
| 37 | 33.39 | Undetermined | 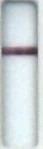 | Undetermined | 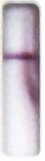 |
| 38 | 35.8 | Undetermined | 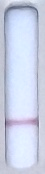 | 40.6 | 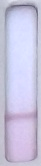 |
| 39 | 36.1 | Undetermined | 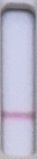 | 38.86 | 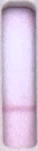 |
| 40 | 36.3 | Undetermined | 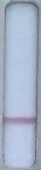 | 41.2 | 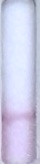 |
| **Group 4: Undetermined, Original Ct > 40, n = 13** | | | | | |
| 41 | 40.2 | Undetermined | 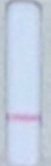 | Undetermined | 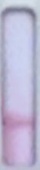 |
| 42 | 41 | 41.15 | 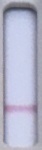 | Undetermined | 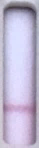 |
| 43 | Undetermined | 40.48 | 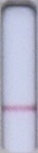 | Undetermined | 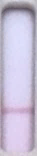 |
| 44 | Undetermined | Undetermined | 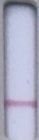 | Undetermined | 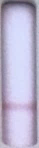 |
| 45 | Undetermined | 40.64 |  | Undetermined |  |
| 46 | Undetermined | Undetermined |  | Undetermined |  |
| 47 | Undetermined | Undetermined |  | Undetermined |  |
| 48 | Undetermined | Undetermined |  | Undetermined |  |
| 49 | Undetermined | Undetermined |  | Undetermined |  |
| 50 | Undetermined | 40.33 |  | Undetermined |  |
| 51 | Undetermined | Undetermined |  | Undetermined |  |
| 52 | Undetermined | Undetermined |  | Undetermined |  |
| 53 | Undetermined | Undetermined |  | Undetermined |  |

**Table S2** Comparison of PCR positive (Ct < 40) and RAT positive rates for clinical samples analyzed by PSAP and conventional methods.

| **Original samples** | **Conventional method**  **w/o enrichment** | | | **PSAP method**  **w/ enrichment** | | |
| --- | --- | --- | --- | --- | --- | --- |
|  | PCR | | RAT | PCR | | RAT |
|  | Positive % | ‾Ct | Positive % | Positive % | ‾Ct | Positive % |
| G1:  Ct < 27  (n = 10) | 6/10=60% | 33.63 | 3/10=30% | 10/10=100% | 32.14 | 9/10=90% |
| G2:  27 < Ct < 31  (n = 16) | 7/16=43.75% | 36.98 | 0/16=0% | 10/16=62.5% | 32.78 | 8/16=50% |
| G3:  31 < Ct < 40  (n = 14) | 1/14=7% | 39.43 | 0/14=0% | 4/14=28.57% | 35.60 | 0/14=0% |
| G4:  Ct > 40  Undetermined (n = 13) | 0/13 = 0% | > 40 | 0/13 = 0% | 0/13 = 0% | > 40 | 0/13=0% |

**Table S3** The elution profile of the LC system.

| Time (min) | %A | %B |
| --- | --- | --- |
| Initial | 98 | 2 |
| 1 | 98 | 2 |
| 12 | 60 | 40 |
| 12.1 | 20 | 80 |
| 13 | 20 | 80 |
| 13.1 | 98 | 2 |
| 15 | 98 | 2 |

**Table S4** The amino acid sequence of peptide fragments used in the mass spectrometry tests.

| Species | Peptide sequence | Parent Ion Found (mass/charge) |
| --- | --- | --- |
| SARS-CoV-2 | DQVILLNK | 471.8 |
| SARS-CoV-2 | LNQLESK | 416.2 |
| SARS-CoV-2 | NPANNAAIVLQLPQGTTLPK | 687.4 |
| Flu A | EGYSLVGIDPFK | 662.8 |
| Flu A | GVFELSDEK | 512.2 |
| Flu B | ILLAATDDK | 480.3 |
| Flu B | TIYFSPIR | 498.8 |
| Flu B | GGGTLVAEAIR | 522.3 |

**Table S5** Geometry parameters of the RAT strips for establishing the 2D models.

| Notation | Value (unit: mm) | Description |
| --- | --- | --- |
| $h_{sp}$ | 0.29 | Sample pad height, as well as the height of the following two pads |
| $h_{wp}$ | 1 | Wick pad height |
| $h_{m}$ | 0.17 | Membrane height |
| $l_{o}$ | 2 | The overlapping length between each part |
| $l_{t}$ | 20 | Test part length |
| $l$ | 80 | Whole test device length |

**Table S6** Theoretical and physical parameters for modeling the 2D liquid flow.

| Dextran concentration wt% % | Viscosity  mPa·s | Surface tension coefficient  mN/m | Surface contact angle  ° | Density  g/cm^3^ |
| --- | --- | --- | --- | --- |
| 0 | 0.38 | 72.8 | 0 | 0.998 |
| 18 | 5.64 | 69.4 | 6.03 | 1.077 |
| 20 | 7.06 | 69.1 | 6.02 | 1.079 |
| 22 | 8.69 | 68.9 | 6.21 | 1.080 |
| 24 | 11.35 | 68.6 | 6.13 | 1.081 |
| 26 | 13.58 | 68.1 | 6.34 | 1.083 |
| 28 | 18.66 | 67.7 | 6.27 | 1.084 |

**Table S7** Theoretical and physical parameters for modeling the 2D liquid flow.

| Notation | Value | Description |
| --- | --- | --- |
| $\epsilon_{p}$ | 0.3104 | Porosity of the test membrane |
| $d_{po}$ | 5 μm | Pore diameter |
| $d_{pa}$= 2.653×$d_{po}$ | 13.265 μm | Porous material particle diameter |
| $\kappa_{s}$ | $\frac{\epsilon_{p}^{3}d_{pa}^{2}}{180{(1-\epsilon_{p})}^{2}}$ | Absolute hydraulic permeability, the Kozeny-Carman model |
| $\theta_{r}$ | 0 | The residual liquid volume fraction |
| $\theta_{s}=\epsilon_{p}$ | 0.3104 | Saturated liquid volume fraction |
| *n*, *l* | 2, 1 | Brooks and Corey's retention model parameter |
| $S_{p}$ | 0 | Storage coefficient |

**Table S8** Simulated results of the time needed for solutions to reach different parts of the test strips.

| Dextran concentration  w/w  Coverage ratio | 18% | 20% | 22% | 24% | 26% | 28% |
| --- | --- | --- | --- | --- | --- | --- |
| 0% | 0 s | 0 s | 0 s | 0 s | 0 s | 0 s |
| 25% | 34 s | 43 s | 54 s | 73 s | 88 s | 127 s |
| 50% | 124 s | 164 s | 202 s | 271 s | 322 s | 503 s |
| 75% | 241 s | 457 s | 565 s | 745 s | 887 s | Flow stops |
| 100% | 699 s | 957 s | Flow stops | Flow stops | Flow stops | Flow stops |

[1] COMSOL, CFD Module User’s Guide, version 6.2. **2023**; pp 661-699.

[2] R. Brooks, Colorado State University, **1965**.

[3] E. Fontes, Modeling a Rapid Detection Test in COMSOL Multiphysics®, <https://www.comsol.com/blogs/modeling-a-rapid-detection-test-in-comsol-multiphysics>, accessed: Feb 23, **2024**.
